# Enhanced SfaTnpB enables single-base-specific, one-pot nucleic acid detection for high-sensitivity diagnostics

**DOI:** 10.1093/nar/gkaf1433

**Published:** 2026-01-08

**Authors:** Bingrong Xu, Sheng Li, Yong Li, Shuhong Zhao, Xinyun Li, Jianlin Han, Di Wu, Shuaicheng Li, Ling Chen, Shengsong Xie, Xiaosong Han, Changzhi Zhao

**Affiliations:** Key Laboratory of Agricultural Animal Genetics, Breeding and Reproduction of Ministry of Education & Key Laboratory of Swine Genetics and Breeding of Ministry of Agriculture and Rural Affairs, Huazhong Agricultural University, Wuhan 430070, P.R. China; Yazhouwan National Laboratory, Sanya 572024, P.R. China; Key Laboratory of Agricultural Animal Genetics, Breeding and Reproduction of Ministry of Education & Key Laboratory of Swine Genetics and Breeding of Ministry of Agriculture and Rural Affairs, Huazhong Agricultural University, Wuhan 430070, P.R. China; Yazhouwan National Laboratory, Sanya 572024, P.R. China; Key Laboratory of Agricultural Animal Genetics, Breeding and Reproduction of Ministry of Education & Key Laboratory of Swine Genetics and Breeding of Ministry of Agriculture and Rural Affairs, Huazhong Agricultural University, Wuhan 430070, P.R. China; Yazhouwan National Laboratory, Sanya 572024, P.R. China; The Cooperative Innovation Center for Sustainable Pig Production, Huazhong Agricultural University, Wuhan 430070, P.R. China; Hubei Hongshan Laboratory, Frontiers Science Center for Animal Breeding and Sustainable Production, Wuhan 430070, P.R. China; Key Laboratory of Agricultural Animal Genetics, Breeding and Reproduction of Ministry of Education & Key Laboratory of Swine Genetics and Breeding of Ministry of Agriculture and Rural Affairs, Huazhong Agricultural University, Wuhan 430070, P.R. China; The Cooperative Innovation Center for Sustainable Pig Production, Huazhong Agricultural University, Wuhan 430070, P.R. China; Hubei Hongshan Laboratory, Frontiers Science Center for Animal Breeding and Sustainable Production, Wuhan 430070, P.R. China; Yazhouwan National Laboratory, Sanya 572024, P.R. China; Department of Computer Science, City University of Hong Kong, Hong Kong 999077, P.R. China; Department of Obstetrics and Gynecology, Peking University Shenzhen Hospital, Shenzhen 518036, P.R. China; Department of Computer Science, City University of Hong Kong, Hong Kong 999077, P.R. China; Department of Pathology, Tianjin Central Hospital of Gynecology and Obstetrics, Tianjin 300100, P.R. China; Key Laboratory of Agricultural Animal Genetics, Breeding and Reproduction of Ministry of Education & Key Laboratory of Swine Genetics and Breeding of Ministry of Agriculture and Rural Affairs, Huazhong Agricultural University, Wuhan 430070, P.R. China; The Cooperative Innovation Center for Sustainable Pig Production, Huazhong Agricultural University, Wuhan 430070, P.R. China; Hubei Hongshan Laboratory, Frontiers Science Center for Animal Breeding and Sustainable Production, Wuhan 430070, P.R. China; Yazhouwan National Laboratory, Sanya 572024, P.R. China; Key Laboratory of Agricultural Animal Genetics, Breeding and Reproduction of Ministry of Education & Key Laboratory of Swine Genetics and Breeding of Ministry of Agriculture and Rural Affairs, Huazhong Agricultural University, Wuhan 430070, P.R. China; Yazhouwan National Laboratory, Sanya 572024, P.R. China

## Abstract

CRISPR/Cas12-based nucleic acid detection has revolutionized molecular diagnostics but shows limited single-nucleotide specificity, limited high-fidelity subtype discrimination, and limited compatibility with one-pot assays, restricting its broader clinical application. Here, we report a transposon-associated transposase B (TnpB) ortholog, SfaTnpB, with high *trans*-cleavage activity, robust single-base mismatch discrimination, and broad temperature tolerance. By stepwise engineering of its guide RNA (ωRNA), we developed an enhanced SfaTnpB (enSfaTnpB) system with markedly improved *trans*-cleavage efficiency. In combination with a TAM-independent split-activator strategy, this system enables precise detection of single-nucleotide polymorphisms. We further developed TOPIC (TnpB-based One-Pot nucleIC acid detection), a one-pot detection platform coupling enSfaTnpB with recombinase-aided amplification (RAA) or loop-mediated isothermal amplification that enables ultrasensitive detection of human papillomavirus (HPV) subtypes 16 and 18 (∼4 copies/μl) and African swine fever virus DNA (∼3 copies/μl). Finally, RAA-TOPIC accurately detected and genotyped 14 high-risk HPV subtypes with high-fidelity subtype discrimination, showing complete concordance with quantitative real-time PCR-based clinical diagnostics. These findings establish TOPIC as a compact, programmable, and scalable molecular detection tool with broad potential for precision diagnostics and point-of-care testing, particularly in resource-limited settings.

## Graphical Abstract

**Graphical Abstract:**
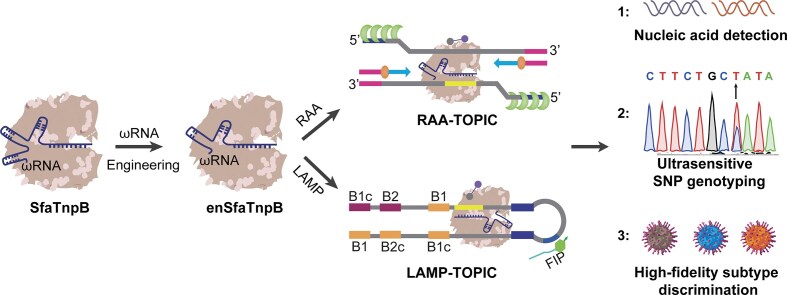

## Introduction

Point-of-care testing (POCT) plays an increasingly vital role in modern infectious disease surveillance, especially in public health and epidemic control, rapid diagnosis of common infections, remote or resource-limited settings, on-site animal disease screening, and home-based healthcare [1–4]. An ideal POCT platform should be sensitive, user-friendly, rapid, robust, equipment-independent, and cost-effective, enabling accurate "sample-to-answer" detection [5]. Nevertheless, current POCT methods still face considerable challenges in attaining single-base resolution, accurate pathogen subtyping, and integrated one-pot detection without compromising sensitivity.

The development of portable nucleic acid detection platforms that provide high sensitivity, single-base specificity, and operational simplicity represents a critical frontier in POCT. It may ultimately enable decentralized pathogen surveillance and precision disease management. Although quantitative real-time PCR (qPCR) assays continue to serve as the gold standard for nucleic acid detection, qPCR-based approaches depend on sophisticated instruments, specialized training, and centralized laboratory infrastructure, consequently restricting their deployment in resource-limited regions, where rapid pathogen identification is often most urgently needed [6–8]. Additionally, qPCR assays have limited adaptability for fluorescence-based readouts, typically detect only a limited number of targets per assay, and generally lack the single-base specificity necessary to distinguish pathogenic strains in multiplexed fluorescence detection applications [9, 10]. This limitation is particularly consequential for primary healthcare systems, where the ability to distinguish high-risk pathogenic variants, such as oncogenic human papillomavirus (HPV) subtypes critical for cervical cancer prevention, could dramatically improve patient outcomes through immediate clinical decision-making [11].

Recent advances in CRISPR-based diagnostics have partially addressed these challenges by harnessing the programmable *trans*-cleavage activity of Cas12a in high-sensitivity detection platforms such as DETECTR (DNA endonuclease-targeted CRISPR trans reporter) [12], HOLMES (one-HOur Low-cost Multipurpose highly Efficient System) [13], and RAVI-CRISPR (RApid VIsual CRISPR) [14]. These systems have demonstrated remarkable capabilities for detecting epidemic pathogens and identifying single-nucleotide polymorphisms (SNPs) with purported attomolar sensitivity. However, their multi-step operational requirements require sequential pre-amplification steps followed by separate CRISPR detection phases, thus introducing procedural complexity and the risk of aerosol contamination, which compromise their suitability for implementation in POCT, particularly in settings where technicians with specialized expertise may be scarce [15].

To overcome these operational barriers, several one-pot detection platforms have emerged that integrate isothermal amplification with CRISPR detection in single-tube assays [16, 17]. For instance, SHINE (Streamlined Highlighting of Infections to Navigate Epidemics) is a relatively sensitive diagnostic tool that combines recombinase polymerase amplification (RPA) with Cas13-based target recognition in a single-step detection assay for SARS-CoV-2 RNA [18]. SHINEv2 further improved this approach by introducing a fast, ambient-temperature sample lysis step, eliminating the need for cold storage [19]. However, one-pot methods are often achieved at the expense of the sensitivity provided by two-step approaches. An innovative RPA-Cas12a-based one-pot detection method was recently developed using suboptimal protospacer-adjacent motifs (PAM), slowing the kinetics of Cas12a-mediated *trans*-cleavage of single-strand DNA (ssDNA) reporters and *cis*-cleavage of targets [20]. Alternatively, the Cas12b PAM-interacting domain can be engineered to reduce interference with loop-mediated isothermal amplification (LAMP)-based target amplification, as in a recently reported LAMP-Cas12b-based one-pot detection method [21]. The SURVEY (heparin Sodium Used for Rapid Viral dEtection and analYsis) one-pot detection platform was developed by modulating Cas12a *cis*-cleavage activity with varying heparin sodium concentrations [22]. While these methods can accurately detect pathogens and reduce the risk of aerosol contamination, they may compromise sensitivity, pose operational challenges, and require further optimization of CRISPR-associated nucleases (Cas) proteins and CRISPR RNA (crRNA) engineering for different targets. It also warrants mention that existing one-pot platforms demonstrate limited capacities for single-base and subtype discrimination due to the low specificity of Cas12a, hampering their broader adoption. These varying drawbacks highlight the urgent need for adaptable, high-sensitivity one-pot detection platforms capable of discriminating single-base variations for genotyping and high-fidelity subtype identification.

The IS200/IS605 transposon family protein, transposon-associated transposase B (TnpB), has recently been identified as the evolutionary ancestor of Cas12 and remains the smallest RNA-guided nuclease reported to date [23–26]. A right-end element (re)RNA guides TnpB recognition of transposon-associated motifs (TAMs) to mediate cleavage of complementary target sequence at the 3′ end of the reRNA via its RuvC domain [27–29]. Previous work has detected *trans*-cleavage activity of TnpB activated by ssDNA or double-stranded DNA (dsDNA) substrates [30, 31]. In particular, TnpB from *Sulfolobus islandicus* exhibits enhanced *trans*-cleavage activity at high temperatures, potentially offering superior compatibility with isothermal amplification conditions [30]. However, its strict dependence on high-temperature activation restricts broader diagnostic applicability. Despite the identification of numerous TnpB systems in various prokaryotic genomes, their *trans*-cleavage activities remain relatively unexplored, with scarce comparisons to Cas12a. Additionally, the sensitivity and specificity of TnpB-based detection platforms are not well understood, posing an obstacle to the development of TnpB-based one-pot nucleic acid detection diagnostic platforms.

In this study, through screening >60 million metagenome-assembled genomes (MAGs), we identified the uncharacterized TnpB ortholog, SfaTnpB (369 amino acids), from *Savagea faecisuis * (*S. faecisuis*). Based on prior TnpB studies, we predicted its TAM and reRNA [31]. Functional assays with ssDNA or dsDNA substrates demonstrated that SfaTnpB exhibits significantly stronger *trans*-cleavage activity than previously reported TnpB orthologs [24, 31]. Further characterization revealed that the SfaTnpB system exhibits high sensitivity and markedly greater specificity than LbCas12a in single-nucleotide discrimination. Through structural modeling with AlphaFold3 [32], we engineered ωRNA variants that substantially improved *trans*-cleavage activity of the enhanced SfaTnpB (enSfaTnpB), which subsequently outperformed LbCas12a. To expand its application, we established a rapid, TAM-independent SNP genotyping workflow using a “split-activator” strategy with enSfaTnpB. In addition, we developed one-pot nucleic acid detection platforms based on RAA and LAMP, enabling rapid, sensitive detection of HPV and African swine fever virus (ASFV). Using this TOPIC (TnpB-based One-Pot nucleIC acid detection) system, we constructed a high-fidelity subtype-discrimination assay capable of simultaneously and rapidly genotyping 14 high-risk human papillomavirus (HR-HPV) subtypes. Validation with 28 clinical samples confirmed that RAA-TOPIC could accurately distinguish all 14 subtypes, highlighting the platform’s potential for clinical diagnostics and population-scale epidemiological surveillance.

## Materials and methods

### Ethical statement

The research on HPV testing using samples from a cervical cancer prevention study at Peking University Shenzhen Hospital (PUSH) has been approved by the Ethics Committee of PUSH (IRB code PUSH [2023]-016).

### Bioinformatic pipeline for mining novel TnpB orthologs

To systematically identify novel TnpB orthologs from microbial genomes, we employed a previously established bioinformatic pipeline [31], as detailed below. A total of 65 224 287 metagenomic contigs were collected from the JGI Integrated Microbial Genomes (IMG), the European Nucleotide Archive (ENA), and the Ocean Microbiomics Database (OMD). In parallel, 107 pairs of left-end (LE) and right-end (RE) sequences of IS605-family transposable elements (TEs) were obtained from the ISfinder database. We employed BLASTn (parameters: -word_size 11 -evalue 10 -gapopen 5 -gapextend 2) to screen metagenomic contigs for candidate TEs harboring both LE and RE sequences. Within the detected LE–RE intervals, open reading frames (ORFs) were predicted using Prodigal (v2.6.3) and translated into protein sequences. Based on previously characterized TnpB proteins, proteins exceeding 300 amino acids were generally retained as putative TnpB orthologs [24, 30, 31, 33]. To distinguish TnpB from TnpA within these genetic elements, we considered both protein length and genomic organization. TnpB proteins are generally longer than 300 amino acids, while TnpA proteins are typically under 200 amino acids. If a single protein is encoded between the transposable elements, it is classified as TnpB; if two proteins are present, the larger one is assigned as TnpB. To remove redundancy, sequences were clustered at 90% identity using CD-HIT (v4.8.1), yielding a non-redundant TnpB protein dataset.

Protein domain architecture was analyzed using the HH-suite package (v3.3.0). Briefly, HHblits was employed to search against the UniRef30 database (https://uniclust.mmseqs.com/) to generate profile HMMs, which were subsequently queried against the Pfam database using HHsearch (https://wwwuser.gwdguser.de/∼compbiol/data/hhsuite/databases/hhsuite_dbs/).

Canonical TnpB proteins were defined by the presence of three conserved domains: helix–turn–helix (HTH), OrfB_IS605, and OrfB_Zn_ribbon. Multiple sequence alignment of the newly identified TnpB orthologs and 78 reported representatives was performed using MAFFT (v7.490), with a focus on the conservation of ten functionally critical residues (N31, G179, D181, E265, L267, C332, C335, C351, C354, and D361). A maximum-likelihood phylogenetic tree was constructed with FastTree (v2.1.11) and visualized on the iTOL online platform (https://itol.embl.de/).

### Protein expression and purification

The candidate TnpB protein sequences were codon-optimized for *Escherichia coli* expression and synthesized by GenScript Biotech (Nanjing, China). A Twin-Strep-tag and a TEV protease cleavage site were fused to the N-terminus of the protein, and the resulting construct was cloned into the pET-28a expression vector via NcoI/XhoI restriction sites, replacing the vector’s native 6× His tag to generate the recombinant TnpB expression plasmids. The plasmids were transformed either alone or together with the corresponding independent driver pCDFDuet-1-ωRNA plasmid into Rosetta (DE3) pLysS competent cells (Weidibio, Cat. No. EC1015M, China), and plated on LB agar containing the appropriate antibiotics. After overnight incubation at 37°C, a single colony was inoculated into 10 ml Terrific Broth (TB) medium containing the appropriate antibiotics for overnight growth. The inoculated culture was subsequently transferred to 2 l of TB medium supplemented with the specified antibiotics. Cultures were grown at 37°C with shaking at 220 rpm until an OD_600_ of 0.6–0.8, then induced with 0.5 mM IPTG (Merck, Cat. No. 420 322, USA) at 16°C with shaking at 180 rpm for 16 h.

Cells were harvested by centrifugation at 12 000 × *g* for 10 min at 4°C, and the pellet was resuspended in lysis buffer (300 mM NaCl, 5 mM HEPES, 1 mM TCEP, 1 mM PMSF, pH 7.5). Cells were lysed by sonication (SCIENTZ, China), and the lysate was clarified by centrifugation at 12 000 × *g* for 1 h at 4°C. The supernatant was filtered through a 0.22-μm membrane prior to purification. All purification steps were performed at 4°C according to IBA Lifesciences protocols. The clarified lysate was loaded onto a pre-equilibrated Strep-Tactin^®^XT 4Flow^®^ column (IBA Life Sciences, Cat. No. 2-5998-000, Germany). The column was washed with five column volumes of Buffer W (IBA Life Sciences, Cat. No. 2-1003-100, Germany), and eluted with six sequential 0.5-column-volume fractions of Buffer BXT (IBA Life Sciences, Cat. No. 2-1045-250, Germany). The column was regenerated using six column volumes of Buffer XT-R, followed by eight volumes of Buffer W. Elution fractions were analyzed by sodium dodecyl sulfate–polyacrylamide gel electrophoresis. Fractions with high purity were pooled and concentrated using an Amicon Ultra centrifugal filter unit (Merck, Cat. No. UFC500396, USA). The protein was dialyzed overnight at 4°C against storage buffer (500 mM NaCl, 20 mM sodium acetate, 0.1 mM EDTA, 0.1 mM TCEP, 50% glycerol, pH 6.0), aliquoted, and stored at −80°C. The full amino acid sequence of TnpB orthologs and its codon-optimized version for *E. coli* is provided in Supplementary Table S1.

### Guide RNA preparation

All DNA templates encoding ωRNA or sgRNA scaffolds were synthesized by GenScript. To generate guide RNAs, *in vitro* transcription was carried out using T7 RNA polymerase as follows: DNA templates were first amplified by PCR using a forward primer harboring the T7 promoter sequence and a reverse primer containing the target-specific region. The resulting PCR products served as templates for *in vitro* transcription using the HiScribe^TM^ T7 High Yield RNA Synthesis Kit (NEB, Cat. No. E2040S, USA) according to the manufacturer’s instructions. For the preparation of separate scaffold and spacer RNAs, the same procedure was applied.

After transcription, the reaction mixtures were treated with DNase I (NEB, Cat. No. M0303S, USA) at 37°C for 15 min to remove template DNA. Transcribed RNAs were purified using the Monarch^®^ RNA Cleanup Kit (NEB, Cat. No. T2050L, USA), and RNA concentrations were determined using a NanoDrop 8000 spectrophotometer (Thermo Fisher Scientific). Finally, purified RNA aliquots were stored at −20°C for downstream applications. All targeting guide sequences used in this study are listed in Supplementary Table S2, and the corresponding ωRNA scaffold sequences are provided in Supplementary Table S3.

### Preparation of DNA templates

Gene fragments encoding ASFV-p72, CSFV-E2, and the L1 capsid protein of high-risk HPV subtypes were synthesized and individually cloned into the pUC57 vector. The *FecB* and *GAPDH* gene fragments were amplified from sheep genomic DNA (gDNA) via PCR and subsequently cloned into the pMD^TM^18-T vector (Takara Bio Inc., Cat. No. 6011, Japan) using a T-A cloning strategy. Unless otherwise specified, all other DNA templates were generated by PCR amplification, and the resulting plasmids were used as templates for downstream detection assays.

Blood samples collected from sheep were used for *FecB* genotyping. Clinical samples for ASFV detection were provided by Hunan Sante Biotechnology Co., Ltd. In contrast, 14 HR-HPV clinical samples consisted of ThinPrep cytology test cell preservation fluid supplied by Peking University Shenzhen Hospital. Nucleic acids were extracted by mixing 50 μl QuickExtract^TM^ DNA Extraction Solution (Lucigen, Cat. No. QE0905T, USA) with each sample, followed by incubation at 65°C for 10 min and 95°C for 5 min. The extracted nucleic acids were stored at −20°C for subsequent experiments.

### *Trans*-cleavage assays

*In vitro trans*-cleavage activity of TnpB was assessed in a standard 20 μl reaction containing reaction buffer, TnpB protein, ωRNA, target DNA, and a reporter probe. For the *trans*-cleavage activity assay, the reaction mixture consists of 1× rCutSmart™ Buffer (NEB, Cat. No. B6004V, USA), 300 nM TnpB, 250 nM ωRNA, 200 nM target DNA, and 250 nM FAM-labelled reporter probe. Reactions were incubated at 37°C for the indicated time, then terminated by adding an equal volume of stop buffer (8 M urea, 0.5 M EDTA). Products were resolved by 20% denaturing polyacrylamide gel electrophoresis and visualized using a chemiluminescence imaging system (Sagecreation, ChampChemi 610).

For fluorescence-based detection, reactions (20 μl total volume) contained 2 μl of 10× reaction buffer, 300 nM TnpB, 900 nM ωRNA, 200 nM target DNA, and 250 nM fluorophore quencher-labeled ssDNA (ssDNA-FQ) reporter (5′-ROX/GTATCCAGTGCG/BHQ2-3′). Reactions were terminated by heating at 98°C for 2 min, diluted with 80 μl of ddH_2_O, and transferred to a black 96-well plate. Fluorescence was measured using a BioTek Synergy H1 microplate reader (excitation: 576 nm, emission: 601 nm) (BioTek Instruments, Inc., USA).

To optimize the *trans*-cleavage activity of SfaTnpB, we systematically evaluated multiple reaction parameters. These included the SfaTnpB-to-ωRNA molar ratio (ranging from 2:1 to 1:5), compatibility with various commercial buffers, specifically NEBuffer™ 2.1 (B7202S), 3.0 (B7003S), r3.1 (B6003V), 4.0 (B7004S), rCutSmart™ (B6004V), and FastDigest Buffer (Thermo Scientific™, B64), temperature sensitivity across 16–55°C, and reporter sequence preference using poly-A, -T, -G, and -C substrates. We further characterized the system by assessing the effect of ωRNA–target pairing length (4–20 nt), TAM sequence tolerance through single-base mismatches, and single-nucleotide discrimination capability across the 20-nt target region.

### Engineering of ωRNA guided by AlphaFold3

To optimize ωRNA design for enhanced SfaTnpB *trans*-cleavage activity, we first evaluated the impact of 5′ truncations based on prior characterizations of native TnpB ωRNA. Guided by the predicted tertiary structure of the SfaTnpB–ωRNA–DNA complex generated using AlphaFold3 (https://alphafoldserver.com), along with secondary structure predictions from UNAfold (https://www.unafold.org/), we systematically truncated the 5′ region to identify the optimal length for efficient activity. Subsequently, sequential deletions were introduced into each stem-loop region of the ωRNA scaffold to assess their individual contributions to SfaTnpB *trans*-cleavage efficiency. The most effective deletion variants were then combined to generate a final engineered ωRNA scaffold. All ωRNA scaffold variant sequences are listed in Supplementary Table S3.

### Split activator and SfaTnpB-mediated nucleic acid assays

Reactions were carried out in a final volume of 20 μl containing 300 nM SfaTnpB, 900 nM ωRNA, and 500 nM DNA activator in 1× FastDigest Buffer supplemented with 1 mM l-proline (Sigma–Aldrich). The mixture was pre-incubated at 37°C for 15 min, followed by the addition of 250 nM ssDNA-FQ reporter (5′-ROX/GTATCCAGTGCG/BHQ2-3′) and the target nucleic acid (ssDNA, dsDNA, or RNA). The reaction was performed at 37°C for 30 min and subsequently inactivated by heating at 98°C for 2 min. To systematically investigate activator properties, we examined the impact of varying activator lengths and target complementarity regions under identical reaction conditions. After termination, the reaction mixture was diluted to a final volume of 100 μl, transferred to a black 96-well plate, and fluorescence was measured using a BioTek Synergy H1 multimode microplate reader (excitation/emission: 576/601 nm). All DNA activator sequences used in this study are listed in Supplementary Table S4.

### One-pot RAA-TOPIC nucleic acid detection platform

The one-pot RAA-TOPIC assay integrates RAA with SfaTnpB-mediated cleavage in a single isothermal reaction system. The reaction was assembled as follows: each 50 μl RAA premix (Qitian Biotech Co., Cat. No. B00000A-48, China) contained 25 μl of Buffer V, 2 μl each of 10 μM forward and reverse primers, one lyophilized enzyme pellet, and 5 μl of Mg(OAc)_2_. For the one-pot reaction, 19.3 μl of RAA mixture was used with 250 nM SfaTnpB, 700 nM ωRNA, 250 nM ssDNA-FQ reporter (5′-ROX/GTATCCAGTGCG/BHQ2-3′), and the corresponding nucleic acid target in a final volume of 25 μl. Reactions were incubated at 37°C for 40 min and terminated by heating at 80°C for 5 min. Fluorescence signals were detected via two approaches: (i) by direct imaging using a blue-light transilluminator and mobile phone camera or (ii) by dilution to a final volume of 100 μl followed by measurement in a black 96-well plate using a BioTek Synergy H1 multimode plate reader (excitation/emission: 576/601 nm). Alternatively, the undiluted 25 μl reaction mixture was directly incubated at 37°C for 2 h in the plate reader, with shaking every 2 min, and real-time fluorescence acquisition monitored via the ROX channel. The primer sequences used for amplification are provided in Supplementary Table S5.

### One-pot RAA-CRISPR/Cas12a assay

The one-pot RAA-CRISPR/Cas12a assay was performed as previously described [34]. Briefly, the RAA premix consisted of 25 μl Buffer V, 2 μl of 10 μM forward primer, 2 μl of 10 μM reverse primer, one lyophilized RAA enzyme pellet, and 16 μl of nuclease-free water. The Cas12a premix contained 100 nM LbCas12a (NEB, Cat. No. M0653T, USA), 200 nM crRNA, and 1× NEBuffer™ 2.1. To assemble the reaction, 21.5 μl of the RAA premix was combined with the Cas12a premix, template DNA, 250 nM ssDNA-FQ reporter (5′-ROX/GTATCCAGTGCG/BHQ2-3′), and 2.5 μl of Mg(OAc)_2_, resulting in a final reaction volume of 25 μl. The reaction was incubated at 37°C for 40 min. Fluorescence signals were recorded using a BioTek Synergy H1 multimode microplate reader (excitation/emission: 576/601 nm). The primer sequences used for amplification are provided in Supplementary Table S5.

### One-pot LAMP-TOPIC detection platform and optimization

The one-pot LAMP-TOPIC assay integrates loop-mediated isothermal amplification (LAMP) and SfaTnpB-mediated cleavage into a single isothermal reaction system. The reaction mixture contained LAMP primers, Bst 3.0 DNA polymerase (NEB, Cat. No. M0374L, USA), dNTP mix (NEB, Cat. No. N0447L, USA), Isothermal Amplification Buffer (NEB, Cat. No. B0374S, USA), MgSO_4_ (NEB, Cat. No. B1003S, USA), SfaTnpB nuclease, engineered ωRNA, 10× FastDigest Buffer, and an ssDNA-FQ reporter (5′-ROX/GTATCCAGTGCG/BHQ2-3′). The primer sequences used for amplification are provided in Supplementary Table S5.

Initial optimization was conducted using 10³ copies of a GAPDH plasmid as the DNA template. For temperature optimization, 25 μl reactions were assembled containing 2.5 μl LAMP primer mix (final concentrations: 3.6 μM FIP/BIP, 0.5 μM F3/B3, 0.9 μM LF/LB), 1 μl Bst 3.0 polymerase, 3.5 μl dNTP mix, 2.5 μl isothermal amplification buffer, 1.5 μl MgSO_4_, 250 nM SfaTnpB, 750 nM ωRNA, 2.5 μl 10× FastDigest Buffer, 250 nM ssDNA-FQ reporter, and 1 μl plasmid template. Reactions were incubated at temperatures ranging from 50 to 65°C to determine optimal performance.

To assess component compatibility, 2 μl of LAMP amplicons were used in place of the plasmid template to evaluate whether LAMP reaction components interfere with SfaTnpB nuclease activity. Primer concentrations were optimized by titrating the primer mix (final concentrations: 1.6 μM FIP, 1.6 μM BIP, 0.2 μM F3, 0.2 μM B3, 0.4 μM LF, 0.4 μM LB) volume from 0.1 to 2.5 μl while keeping all other components constant. To further minimize potential inhibition caused by Bst polymerase, the total reaction volume was increased to 35 μl. The final optimized 35 μl reaction system included 1.2 μl primer mix, 1 μl Bst 3.0 polymerase, 3.5 μl dNTP mix, 2.5 μl isothermal amplification buffer, 1.5 μl MgSO_4_, 250 nM SfaTnpB, 750 nM ωRNA, 2.5 μl 10× FastDigest Buffer, 250 nM ssDNA-FQ reporter, and the appropriate DNA template. Reactions were carried out at 48°C for 40 min, followed by 80°C for 5 min.

### One-pot LAMP-CRISPR/Cas12b assay

The one-pot LAMP-CRISPR/Cas12b assay was performed as previously described [35], combining loop-mediated isothermal amplification with AapCas12b-mediated detection in a single reaction system. The 25 μl reaction mixture consisted of 2.5 μl Isothermal Amplification Buffer, 6 mM MgSO_4_, 1.4 mM dNTPs, 0.2 μM each of F3 and B3 primers, 1.6 μM each of FIP and BIP primers, 0.4 μM each of LF and LB primers, 150 nM AapCas12b (Shanghai Tolo Biotech Co., Cat. No. 32118, China), 100 nM sgRNA, 250 nM ssDNA-FQ reporter (5′-ROX/GTATCCAGTGCG/BHQ2-3′), 8 U Bst 3.0 DNA polymerase, and the corresponding DNA template. The reactions were incubated at 60°C in a thermostatic metal bath for 50 min. Fluorescence signals were acquired using a BioTek Synergy H1 multimode microplate reader (excitation/emission: 576/601 nm). The primer sequences used for amplification are provided in Supplementary Table S5.

### Genotyping of 14 high-risk HPV subtypes using the one-pot RAA-TOPIC detection system

Complete *L1* gene sequences of 14 HR-HPV subtypes, HPV16, 18, 31, 33, 35, 39, 45, 51, 52, 56, 58, 59, 66, and 68, were retrieved from the NCBI database, with corresponding reference accession numbers: LC368980.1 (HPV16), KC470210.1 (HPV18), OP712005.1 (HPV31), HQ537701.1 (HPV33), MT217174.1 (HPV35), OP971074.1 (HPV39), EF202156.1 (HPV45), OR997935.1 (HPV51), LC270053.1 (HPV52), LR862043.1 (HPV56), LR861973.1 (HPV58), ON365489.1 (HPV59), EF177185.1 (HPV66), and KC470280.1 (HPV68). Multiple sequence alignments were performed using ClustalX2, and subtype-specific TAM regions were designed within the conserved region targeted by the previously reported RPA primers [36]. Candidate TAMs met the following criteria: (i) uniquely present in the target subtype or shared among only a few subtypes and (ii) located within 5–12 nucleotides downstream of the TAM region, containing multiple subtype-specific mutation sites. Based on these regions, ωRNAs specific to each subtype were designed for genotyping. Based on these regions, subtype-specific ωRNAs were designed for precise genotyping. The complete list of ωRNA guide sequences is provided in Supplementary Table S2.

For primary screening of clinical samples, the standard RAA-TOPIC reaction was adapted by including 14 subtype-specific ωRNAs (each at 500 nM) in a single reaction. Positive samples from the initial screen were then subjected to individual genotyping using 14 parallel one-pot reactions, each containing a single subtype-specific ωRNA. All genotyping reactions were performed under identical conditions and system configurations as the primary RAA-TOPIC assay. The primer sequences used for amplification are provided in Supplementary Table S5.

### Sample detection by qPCR

qPCR assays were conducted using the LightCycler^®^ 480 Instrument II system (Roche, Switzerland). For ASFV detection, reactions were performed using NovoStart SYBR High-Sensitivity qPCR SuperMix (Novoprotein Scientific Inc., Cat. No. E099, China) following previously reported protocols [37]. The thermal cycling conditions were initial denaturation at 95°C for 3 min, followed by 45 cycles of 95°C for 5 s and 60°C for 30 s.

For HPV16/18 detection, a TaqMan probe-based qPCR assay was performed as described in previous studies using Luna^®^ Universal Probe qPCR Master Mix (NEB, Cat. No. M3004L, USA) [38]. The amplification protocol consisted of an initial denaturation at 95°C for 3 min, followed by 40 cycles of 95°C for 10 s and 60°C for 30 s. The qPCR primer sequences and TaqMan probe sequences are listed in Supplementary Table S5.

### Statistical analysis

Data from independent experiments are presented as mean ± standard error of the mean (SEM). Statistical significance between experimental and control groups was determined using two-tailed Student’s *t*-tests, conducted in GraphPad Prism version 9.0. Significance thresholds were defined as follows: *P* < .05 (*), *P* < .01 (**), *P* < .001 (***), and *P* < .0001 (****). Differences with *P* ≥ 0.05 were considered not significant (ns).

## Results

### TnpB from *S. faecisuis* exhibited robust *trans*-cleavage activity

We first systematically assessed the *trans*-cleavage activity of previously characterized TnpB orthologs, including ISDra2, ISDge10, ISAam1, and ISYmu1 [24, 31]. We compared their activity, along with that of LbCas12a, toward ssDNA or dsDNA activators using a ssDNA-FQ reporter assay. The results revealed that LbCas12a exhibited the strongest *trans*-cleavage activity, while all tested TnpB orthologs showed negligible activity on both target types (Supplementary Fig. S1). Given that limited *trans*-cleavage activity restricts the diagnostic utility of TnpBs, we aimed to identify additional TnpB variants with higher *trans*-cleavage activity for potential use in nucleic acid detection.

**Figure 1.**
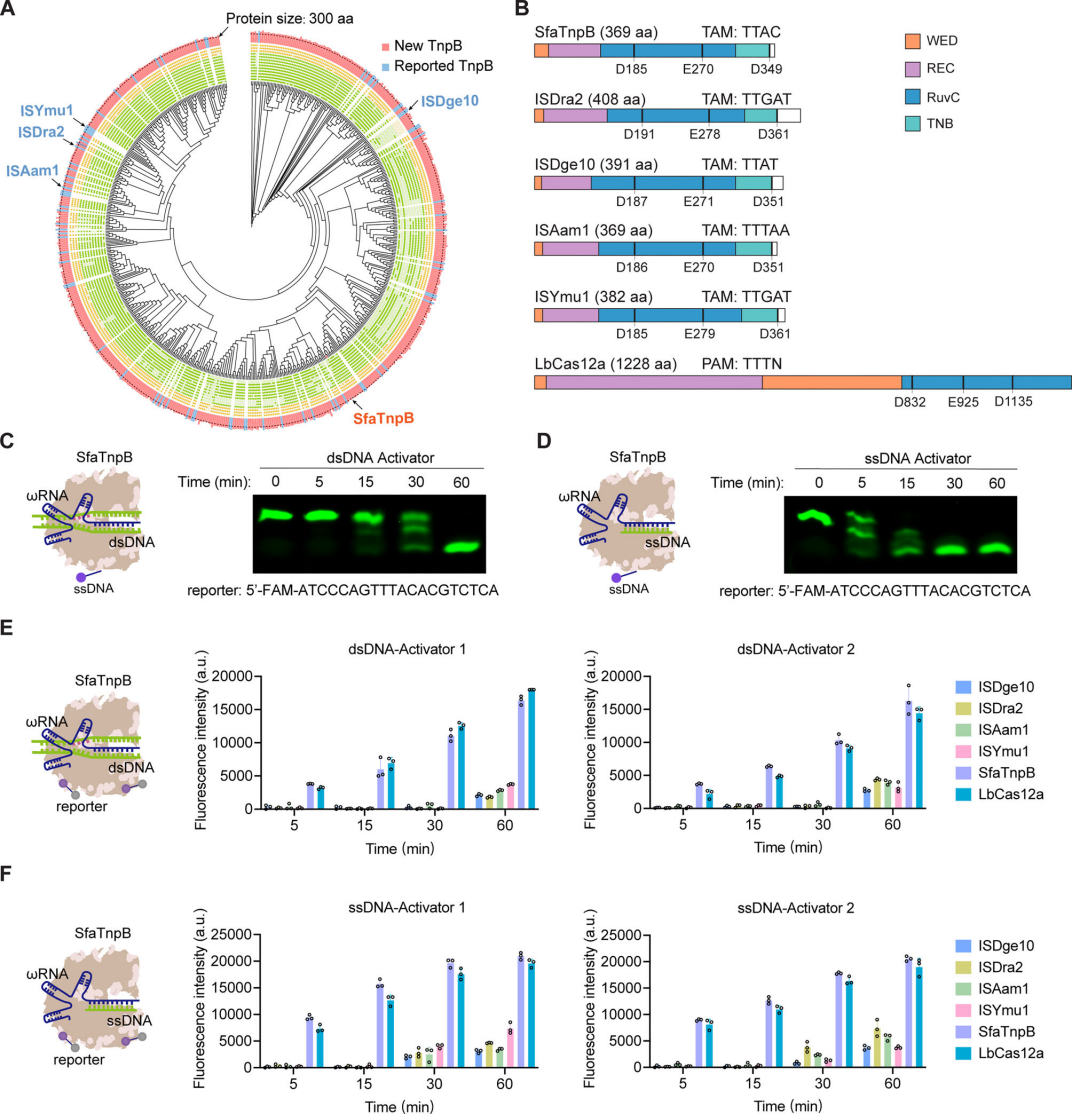
*Trans*-cleavage activity of TnpB from *S. faecisuis*. (**A**) Phylogenetic tree of TnpB orthologs identified by screening the JGI, ENA, and OMD databases and 78 reported TnpBs. The outer track (histogram) illustrates protein lengths. The middle track indicates the presence of structural domains, including (from inner to outer rings): HTH, OrfB_IS605, and OrfB_Zn_ribbon domains. The interior track indicates the presence of conserved functional residues, including (from the center outward) N31, G179, D181, E265, L267, C332, C335, C351, C354, and D361. (**B**) Domain architectures of SfaTnpB, ISDra2, ISDge10, ISAam1, and ISYmu1 TnpBs, as well as LbCas12a. The WED, REC, RuvC, and TNB domains are shown. Protein lengths are drawn to scale. aa, amino acid. Denaturing gel electrophoresis showing *trans*-cleavage of a FAM-labeled ssDNA-FQ reporter by TnpB–ωRNA complexes activated by dsDNA (**C**) or ssDNA activators (**D**). Evaluation of SfaTnpB, ISDra2, ISDge10, ISAam1, ISYmu1 TnpBs, and LbCas12a *trans*-cleavage activity using dsDNA (**E**) or ssDNA (**F**) activators. Data are shown as means ± s.d. (*n* = 3 technical replicates).

We then screened ∼60 million MAGs from the JGI IMG, ENA, and OMD Databases [39–41]. Following a previously reported approach [31], we first extracted the LE and RE sequences from all 107 annotated IS605 family members in the ISfinder database [33]. Using these sequences as queries to search metagenomic contigs, we identified 4 964 sequences containing both LE and RE elements, then used Prodigal software to predict the ORFs between the LEs and REs; putative TnpB proteins were distinguished from TnpA based on sequence length. Clustering analysis of the TnpB sequences at 90% amino acid identity yielded 552 non-redundant TnpB candidates (Fig. 1A). Closer examination of their conserved residues and functional domains revealed that 259 of these proteins harbored the canonical HTH domain, OrfB_IS605, and OrfB_Zn_ribbon domains, and retained 10 functionally critical residues (N31, G179, D181, E265, L267, C332, C335, C351, C354, and D361). We selected nine TnpBs (ISAba30-1, ISTac1, ISAba30-2, ISAba30-3, ISCsa5, ISTel2, IS609, ISBth16, and SfaTnpB) (Supplementary Fig. S2), successfully purified from the 19 representative candidates, to conduct further experimental validation and functional characterization.

Subsequently, the results demonstrated that, among the nine candidate TnpB orthologs assessed for their *trans*-cleavage activity against dsDNA activators, SfaTnpB (369 amino acids) from *S. faecisuis* displayed the highest *trans*-cleavage activity (Fig. 1B and Supplementary Fig. S2). Further assessment of SfaTnpB cleavage efficiency, in complex with ωRNA on a FAM-labeled non-specific ssDNA-FQ reporter, revealed significantly faster substrate cleavage when activated by ssDNA compared to dsDNA (Fig. 1C and D). Benchmarking its *trans*-cleavage activity against LbCas12a and the four previously reported TnpBs (ISDra2, ISDge10, ISAam1, and ISYmu1) using an ssDNA-FQ reporter assay (Fig. 1E and F). Notably, SfaTnpB exhibited the highest activity among TnpB orthologs, comparable to that of LbCas12a. Consistent with prior observations of LbCas12a and our above results, ssDNA triggered its activity more effectively than dsDNA (Supplementary Fig. S3), reinforcing the evolutionary link between TnpBs and type V CRISPR nucleases. Collectively, these results indicated that the SfaTnpB candidate exhibits robust *trans*-cleavage activity, suggesting its potential suitability for nucleic acid detection.

### Characterization of SfaTnpB *trans*-cleavage

**Figure 2.**
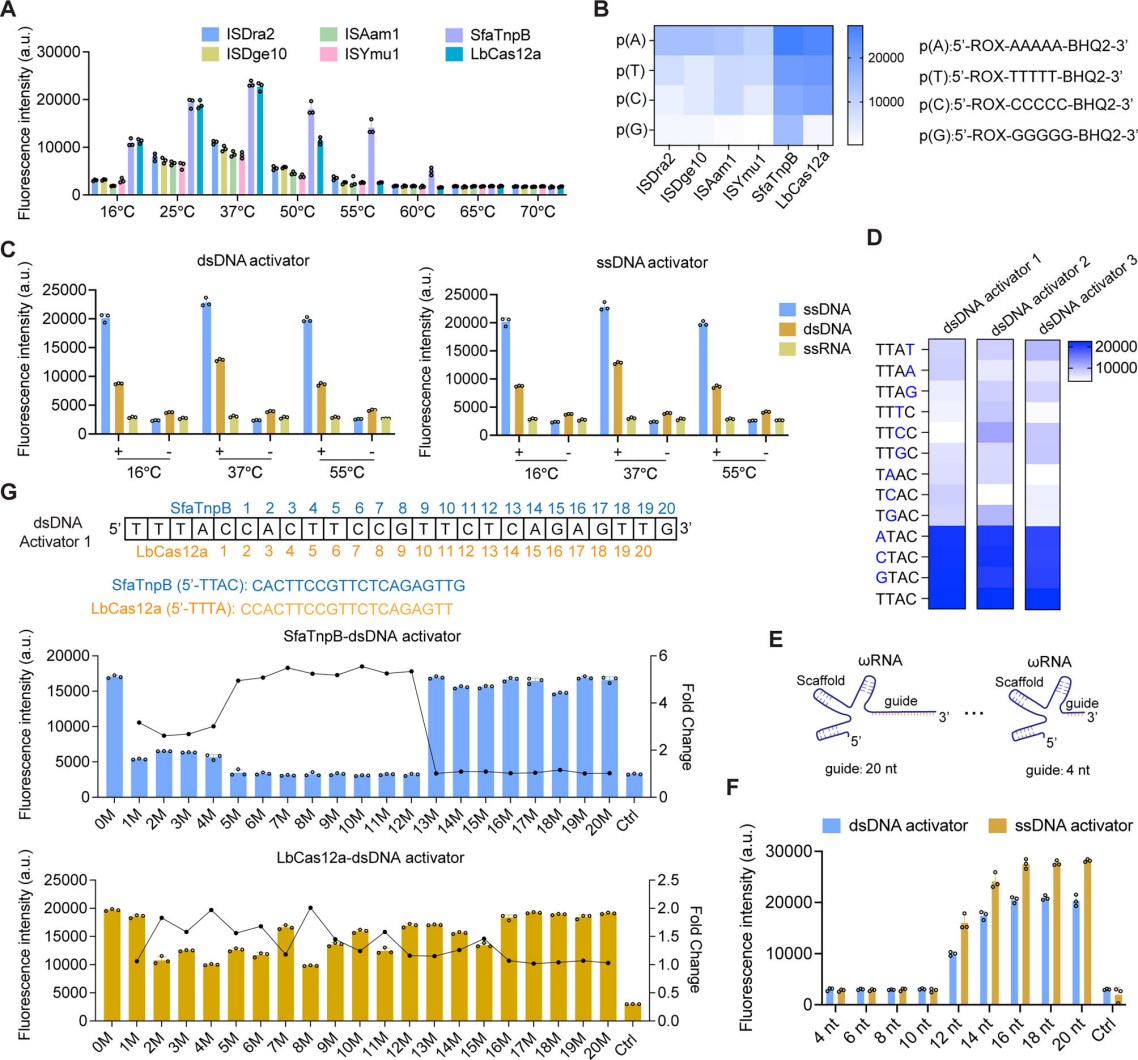
Characterization of SfaTnpB *trans*-cleavage activity. (**A**) Evaluation of *trans*-cleavage activity of SfaTnpB, ISDra2, ISDge10, ISAam1, ISYmu1 TnpBs, and LbCas12a at different temperatures. (**B**) Assessment of preferential sequence contexts for SfaTnpB, ISDra2, ISDge10, ISAam1, ISYmu1 TnpBs, and LbCas12a using an ssDNA-FQ reporter. p (A), poly A; p (T), poly T; p (C), poly C; p (G), poly G. (**C**) Investigation of the reporter preference (ssDNA, dsDNA, or ssRNA) for SfaTnpB-mediated *trans*-cleavage at different temperatures. +, presence of template; −, absence of template. (**D**) Analysis of the TAM sequence requirements for SfaTnpB. (**E**) Scheme for stepwise truncation of the 20-nt ωRNA guide sequence. (**F**) Assessment of *trans*-cleavage activity for each truncated ωRNA. Ctrl, no template control. (**G**) Assessment of single-base discrimination specificity by SfaTnpB and LbCas12a with dsDNA activators. Fold change was calculated by normalizing the activity of the WT to that of each single-nucleotide mismatch (WT/mismatch). Brackets indicate TAM and PAM sequences. 0M, fully matched target; Ctrl, no template control. All data are shown as means ± s.d. (*n* = 3 technical replicates).

To systematically evaluate the *trans*-cleavage capabilities of SfaTnpB, we first examined the effects of different reaction temperatures on TnpB orthologs, comparing them with LbCas12a. SsDNA-FQ reporter assays showed that TnpB orthologs and LbCas12a all exhibited the highest *trans*-cleavage activity at 37°C (Fig. 2A). However, the activity of all four TnpB orthologs and LbCas12a significantly diminished as temperature increased, except for SfaTnpB, which maintained relatively high activity up to 55°C, while its activity decreased substantially at 60°C and became nearly undetectable at 70°C. These results indicated that SfaTnpB could sustain high *trans*-cleavage activity across a broad temperature range, from 16°C to 55°C.

To determine the optimal sequence required for efficient cleavage of ssDNA-FQ reporter, we assessed preferential sequence contexts of TnpB orthologs and LbCas12a using a set of ssDNA-FQ reporters with varying sequences. While the TnpB orthologs and LbCas12a could all efficiently cleave ssDNA-FQ reporters containing poly (A), poly (T), or poly (C) sequences, only SfaTnpB could mediate cleavage of the poly (G)-containing reporter (Fig. 2B). We further evaluated whether secondary structures in the ssDNA-FQ reporter affect SfaTnpB’s *trans*-cleavage activity. Our data showed that SfaTnpB successfully cleaves structured ssDNA-FQ reporters in the presence of both dsDNA and ssDNA activators (Supplementary Fig. S4). However, cleavage efficiency was moderately reduced compared to that observed with linear ssDNA-FQ reporters.

As LbCas12a has previously demonstrated *trans*-cleavage of both dsDNA and ssDNA-FQ reporters [13], we next investigated whether SfaTnpB preferentially catalyzed *trans*-cleavage of ssDNA, dsDNA, or single-stranded RNA (ssRNA) substrates, using both ssDNA and dsDNA activators. The results showed that both ssDNA-FQ and dsDNA-FQ substrates were efficiently cleaved from 16°C to 55°C, while no activity was detected with ssRNA-FQ substrates (Fig. 2C). These results suggested that, excluding ssRNA activators, SfaTnpB exhibits no obvious sequence or substrate biases. Subsequent optimization of reaction components showed that SfaTnpB exhibited its highest *trans*-cleavage activity in reactions in Thermo FastDigest Buffer with a 1:3 molar ratio of SfaTnpB to ωRNA (Supplementary Fig. S5).

Based on the characteristics of the TnpB family, we speculated that SfaTnpB recognized a 5′-TTAC TAM. We subsequently confirmed that this motif is sufficient to trigger *trans*-cleavage activity in the presence of dsDNA activators (Fig. 1C and E). To further investigate the TAM requirements of SfaTnpB, we conducted *trans*-cleavage assays using three different dsDNA activators with mutated TAM sequences. These assays indicated that SfaTnpB could target all 5′-NTAC TAMs, while single-nucleotide mutations at positions 2 to 4 of the TAM sequence resulted in significantly reduced *trans*-cleavage activity (Fig. 2D). Next, we aimed to determine the minimal length of the guide-target matching region required to activate SfaTnpB activity. For these assays, we generated variants of the 20-nt guide sequence with progressive truncations of the 3ʹ terminus (Fig. 2E). SsDNA-FQ reporter assays with each truncated guide indicated that *trans*-cleavage activity required guides 14 nt or longer to maintain efficiency comparable to that with full-length 20-nt guides for both dsDNA and ssDNA activators. While the 12-nt guide resulted in slightly reduced activity, shorter guides (less than 12 nt) resulted in completely abolished activity (Fig. 2F). These results indicated that a minimum of 12 base pairs in the guide RNA-target duplex region was required to support the *trans*-cleavage activity of SfaTnpB.

Because the application of LbCas12a for distinguishing pathogenic strains and SNPs is limited by its low single-base specificity [36, 42, 43], we also evaluated SfaTnpB’s single-base specificity. Using three different dsDNA activators flanked by a 5′-TTAC TAM (for SfaTnpB) or a 5′-TTTA/GTTA PAM (for LbCas12a), we designed 20 dsDNA activator variants, each containing a single-nucleotide mismatch across the 20-nt guide region. Assessment of *trans*-cleavage activity with these dsDNA activators by ssDNA-FQ assays showed that LbCas12a exhibited irregular and inconsistent differences in *trans*-cleavage activity between the variants and the fully matched target. In contrast, SfaTnpB showed significantly reduced *trans*-cleavage activity with dsDNA activators containing a mismatch at positions 5 through 12 (Fig. 2G and Supplementary Fig. S6A). Consistent with the dsDNA results, evaluation using a ssDNA activator showed that mismatches at positions 5–12 similarly caused a marked reduction in *trans*-cleavage activity (Supplementary Fig. S6B), further confirming the high sensitivity of SfaTnpB to single-nucleotide mismatches within this critical region.

Furthermore, we assessed the impact of both the position and the types of 1 to 3 mismatches on the specificity of SfaTnpB. An 18-bp target region was used and divided into six 3-bp groups (G1–G6) (Supplementary Fig. S7A). Single-base mismatches in groups G2–G4 resulted in a marked reduction in fluorescence (Supplementary Fig. S7B and C), consistent with the decreased *trans*-cleavage activity observed for mismatches at positions 5–12 described above. Notably, the fluorescence intensity was largely independent of the identity of the mismatched nucleotide. In addition, 2 to 3 mismatches in groups G1 and G5 led to substantial fluorescence attenuation. By contrast, mismatches in groups G5 and G6 displayed weaker overall attenuation compared with those in G1–G4 (Supplementary Fig. S7B), which aligns with the requirement of only a 12-nt complementary region for SfaTnpB activation. These results indicated that SfaTnpB exhibited greater sensitivity to single-nucleotide mismatch, suggesting high specificity for single-base discrimination within the 5–12 nt region.

We next evaluated the detection sensitivity of the SfaTnpB- and Cas12a-based systems for variant allele frequencies (VAFs) when coupled with RAA. Using the drug-resistant *FLT3* D835Y mutation in acute myeloid leukemia as a model, we prepared wild-type *FLT3* (*FLT3*-WT) and *FLT3*-D835Y plasmids. We mixed them at varying ratios, with the mutant allele constituting 100%, 50%, 25%, 10%, 1%, 0.1%, 0.01%, and 0.001% of total DNA templates. For SfaTnpB detection, RAA primers were designed to introduce an A base, generating a 5′-TAC TAM sequence compatible with ωRNA recognition. For Cas12a, a previously validated mutation-specific crRNA was employed to minimize wild-type cross-reactivity (Supplementary Fig. S8A) [44]. The specificity of the ωRNA targeting D835Y was confirmed before analysis (Supplementary Fig. S8B). Our results demonstrated that LbCas12a detected the D835Y mutation at a VAF of 1%, whereas SfaTnpB exhibited tenfold higher sensitivity, reliably detecting the mutant allele at 0.1% VAF (Supplementary Fig. S8C and D). These results underscore the potential of SfaTnpB for detecting low-frequency mutations and rare genetic variants in molecular diagnostics.

Previous studies of Cas12a have shown that split crRNA constructs enable high sensitivity, multiplex detection of RNA and DNA [45]. To determine whether SfaTnpB could be applied in a similar strategy, we compared the efficiency of ωRNA-scaffold-spacer-directed (split ωRNA) *trans*-cleavage with that of ωRNA-directed (full-length ωRNA) *trans*-cleavage using an ssDNA-FQ reporter with ssDNA or dsDNA activators (Supplementary Fig. S9A). We found that SfaTnpB exhibited robust *trans*-cleavage directed by full-length ωRNA, but no detectable activity occurred with the corresponding split ωRNA (Supplementary Fig. S9B). In contrast, a parallel comparison with LbCas12a confirmed that either ssDNA or dsDNA activators could activate crRNA-scaffold-spacer-directed (split crRNA) and crRNA-directed (full-length crRNA) *trans*-cleavage activities of LbCas12a (Supplementary Fig. S9C and D). Furthermore, neither SfaTnpB nor LbCas12a exhibited detectable *trans*-cleavage activity when directed by the spacer or scaffold alone (Supplementary Fig. S9B and D). Collectively, these results suggested that SfaTnpB requires a complete and intact ωRNA for its *trans*-cleavage function.

### Enhanced *trans*-cleavage activity via engineered ωRNA with AlphaFold3

**Figure 3.**
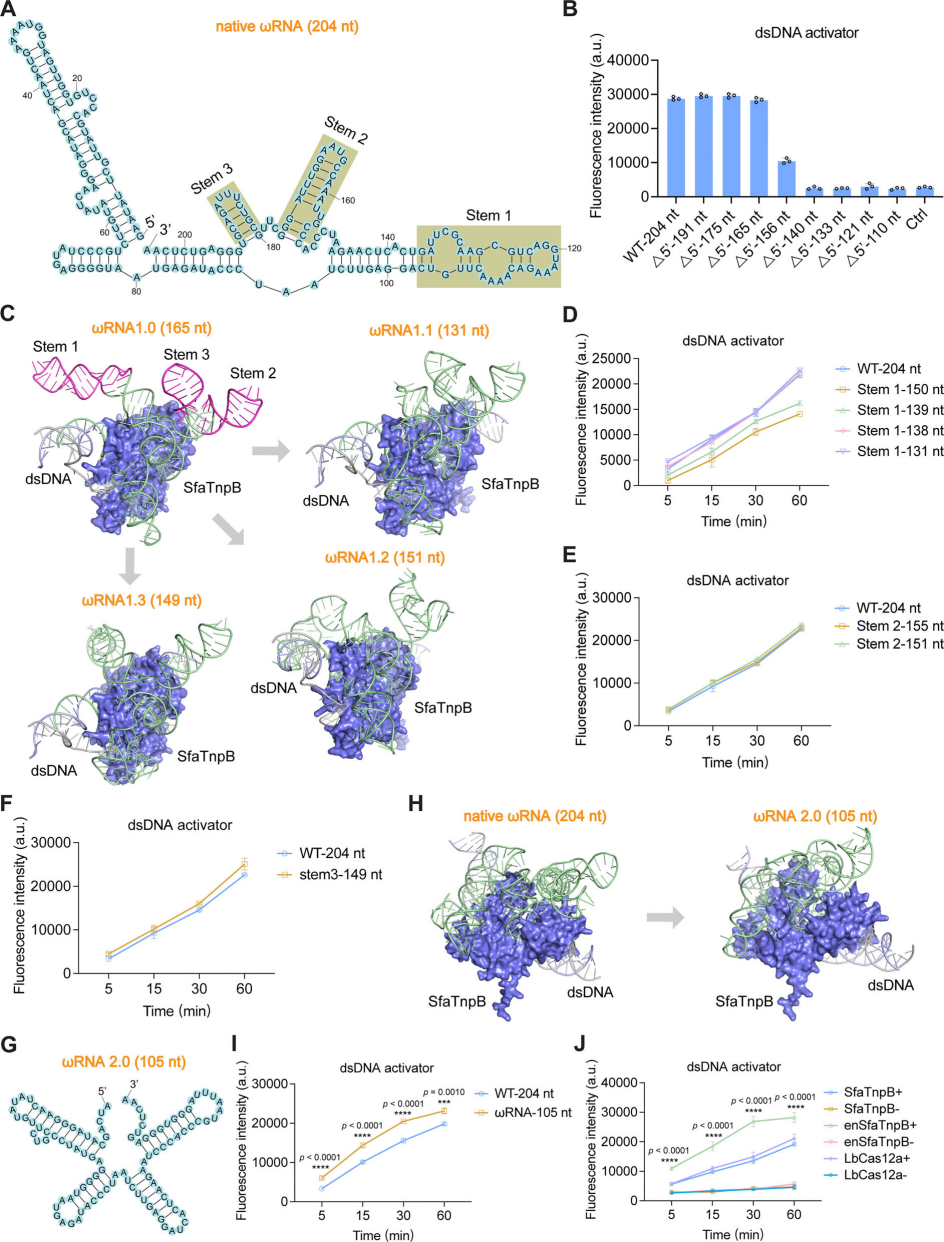
Stepwise engineering of SfaTnpB-associated ωRNA to increase *trans*-cleavage activity. (**A**) Predicted secondary structure of the native ωRNA (204 nt), comprising the Stem1, Stem2, and Stem3 stem-loop regions. (**B**) Assessment of *trans*-cleavage activity using ωRNAs with progressive 5′ truncation and a dsDNA activator. Ctrl, no template control. (**C**) Predicted structures of the SfaTnpB–dsDNA–ωRNA complexes (including ωRNA1.0, ωRNA1.1, ωRNA1.2, and ωRNA1.3), generated by AlphaFold3. Evaluation of the *trans*-cleavage activity by ωRNAs with truncated Stem1 (**D**), Stem2 (**E**), or Stem3 (**F**) regions using a dsDNA activator. (**G**) Predicted secondary structure of ωRNA2.0 (105 nt), featuring four compact stem-loop domains. (**H**) Structures of the SfaTnpB–dsDNA–ωRNA complexes with native ωRNA or ωRNA2.0, predicted with AlphaFold3. (**I**) Comparison of SfaTnpB *trans*-cleavage activity using native ωRNA (WT-ωRNA) versus ωRNA2.0 with a dsDNA activator. (**J**) Comparison of *trans*-cleavage activity between enSfaTnpB and LbCas12a using dsDNA activator. +, presence of template; −, absence of template. All data are shown as means ± s.d. (*n* = 3 technical replicates). Statistical analysis was performed using a two-tailed Student’s *t*-test to compare ωRNA-105 nt with WT-204 nt in panel (I) and the enSfaTnpB + group with the LbCas12a + group in panel (J).

We hypothesized that truncating and optimizing the ωRNA might enhance SfaTnpB *trans*-cleavage activity, as engineering the ωRNA of known ISDra2 TnpB had improved its editing efficiency [25, 26]. To explore this possibility, we used UNAfold [46] to predict the secondary structure of the native ωRNA (WT-ωRNA) scaffold associated with SfaTnpB and design a stepwise truncation strategy (Fig. 3A). Given that truncation of the ωRNA 5′ region reportedly exerted little influence on ISDra2 editing efficiency [25], we progressively truncated the ωRNA 5′ region of SfaTnpB. Subsequent reporter assays with dsDNA or ssDNA activators revealed that a 165 nt ωRNA (ωRNA1.0) allowed comparable activity to that of the 204 nt WT-ωRNA with a dsDNA activator (Fig. 3B and Supplementary Fig. S10A), whereas 133 nt ωRNA (ωRNA-133) maintained efficient SfaTnpB activity when using an ssDNA activator (Supplementary Figs S10B and S11A and B).

Revisiting the predicted secondary structure of the 204-nt ωRNA, we observed that it folds into three distinct stem-loops (Fig. 3A). Further modeling of the TnpB–dsDNA–ωRNA1.0 complex structure with AlphaFold3 suggested that none of the stem-loops interacted with the TnpB protein (Fig. 3C), leading us to hypothesize that these three structures might not participate in SfaTnpB *trans*-cleavage. Activity assays with the ωRNA1.0 variants harboring sequential truncation of stems 1, 2, and 3 revealed that eliminating stem 1 (ωRNA1.1, 131 nt), stem 2 (ωRNA1.2, 151 nt), or stem 3 (ωRNA1.3, 149 nt) resulted in comparable or even enhanced *trans*-cleavage activity compared to that of the WT-ωRNA, using either dsDNA or ssDNA activators (Fig. 3C–F and Supplementary Figs S10C–E and S11C–E).

Secondary structure predictions of the engineered 105-nt ωRNA (ωRNA2.0), containing deletions of the 5′ region, Stem 1, Stem 2, and Stem 3, showed four distinct, compact stem-loop domains (Fig. 3G–H and Supplementary Fig. S10F). Unexpectedly, activity assays revealed that SfaTnpB exhibited significantly stronger *trans*-cleavage with ωRNA2.0 compared to the WT-ωRNA, using either dsDNA or ssDNA activators (Fig. 3I and Supplementary Fig. S11F). We found that combining ωRNA2.0 with the above optimized reaction conditions, including Thermo FastDigest Buffer and a 1:3 molar ratio of SfaTnpB to ωRNA, to generate an enhanced SfaTnpB system (enSfaTnpB) resulted in markedly enhanced *trans*-cleavage activity, surpassing that of LbCas12a (Fig. 3J). Collectively, these results indicated that the ωRNA of SfaTnpB could be engineered through sequence truncation to enhance *trans*-cleavage activity, thereby establishing an improved enSfaTnpB-based detection platform.

### Ultrasensitive detection of single-point mutations by enSfaTnpB

**Figure 4.**
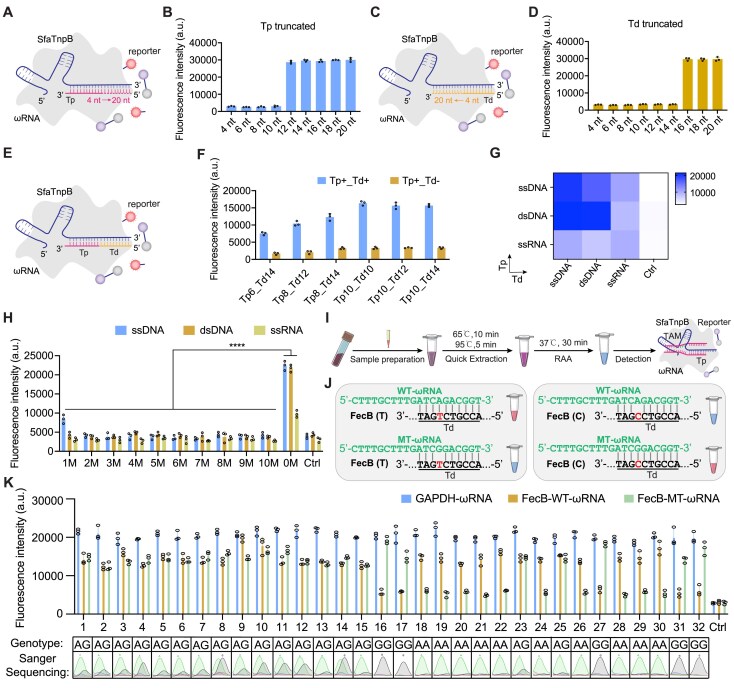
Sensitivity and specificity of SfaTnpB in SNP detection. (**A**) Schematic of Tp activator recognition by the SfaTnpB–ωRNA complex. (**B**) Assessment of *trans*-cleavage activity triggered by truncated Tp activators. (**C**) Schematic of Td activator recognition by the SfaTnpB–ωRNA complex. (**D**) Assessment of *trans*-cleavage activity triggered by truncated Td activators. (**E**) Schematic of enSfaTnpB-ωRNA complex activation by two split-activators. (**F**) Comparison of *trans*-cleavage activities induced by different combinations of Tp and/or Td activators of varying lengths. +, presence of template; −, absence of template. (**G**) Heatmaps of enSfaTnpB *trans*-cleavage activity triggered by different combinations of ssDNA, TAM-containing dsDNA, or ssRNA Tp, as well as ssDNA, dsDNA, or ssRNA Td activators. Ctrl, no template control. (**H**) Assessment of enSfaTnpB *trans*-cleavage activity using ssDNA, dsDNA, or ssRNA Td activators harboring single-base mismatches. 0M, fully matched target; Ctrl, no template control. Statistical analysis was performed using a two-tailed Student’s *t*-test. ^****^P < .0001. (**I**) Schematic for SNP genotyping with a split-activator-based enSfaTnpB system incorporating RAA. (**J**) Diagram of target wild-type and mutant *FecB* (A-to-G/T-to-C) DNA sequences with corresponding Td sequences. Td activator sequence is underlined. (**K**) Genotyping of 32 sheep blood samples using the split-activator-based enSfaTnpB system coupled with RAA. Ctrl, no template control. All data are shown as means ± s.d. (*n* = 3 technical replicates).

Given the high specificity of SfaTnpB for discriminating mismatched single bases at positions 5 to 12 of the ωRNA-guide region, we next sought to harness this feature for rapid SNP genotyping. However, the strict requirement for a 5′-NTAC TAM sequence flanking the target site substantially limited its potential applicability. To overcome this constraint, we explored “split-activator” strategies, which can boost Cas12a *trans*-cleavage activity and enhance the specificity of PAM-independent mutation detection [47]. To test whether this approach could reduce enSfaTnpB’s TAM dependency, we first evaluated the minimum possible length of ssDNA activators complementary to either the TAM-proximal (Tp) or TAM-distal (Td) ωRNA regions (Fig. 4A–D). The results demonstrated that *trans*-cleavage was abolished entirely with Tp activators <12 nt or Td activators <16 nt. We then systematically assessed combinations of sub-threshold Tp and Td activators to identify the optimal split configuration. Notably, combining a 10-nt Tp activator and a 10-nt Td activator yielded the strongest *trans*-cleavage activity (Fig. 4E and F). These findings indicated that enSfaTnpB could tolerate split ssDNA activators and still mediate efficient *trans*-cleavage.

To assess the versatility of this system, we next tested enSfaTnpB with various combinations of ssDNA, dsDNA, or ssRNA Tp and Td activators (Fig. 4G). We observed that the *trans*-cleavage activity could be efficiently triggered when the Tp activator was supplied as ssDNA, dsDNA, or ssRNA, and the Td activator was supplied in any of these forms, although ssRNA Tp activators were associated with obviously lower activity compared to ssDNA or dsDNA counterparts. To further evaluate specificity, we introduced single-base mismatches in the Td activator across different substrates. We observed that mismatches at positions 1–10 of dsDNA or ssRNA Td activators, or positions 2–10 of ssDNA Td activators, almost completely abolished *trans*-cleavage activity (Fig. 4H). Together, these results confirmed that a split-activator strategy could significantly enhance enSfaTnpB’s capacity to discriminate mismatches, enabling precise TAM-independent SNP detection.

As a proof-of-principle practical application of the split-activator-based enSfaTnpB system for rapid SNP genotyping, we integrated our system with RAA in a rapid detection workflow (Fig. 4I). We selected a well-characterized A-to-G/T-to-C point mutation at the *BMPR1B* locus (g.A746G, p.Q249R, also known as *FecB*), strongly associated with sheep fertility [48], as a model target. To assess allele-specific detection, we applied both wild-type (WT-ωRNA) and mutant (MT-ωRNA) guides (Fig. 4J). Using crude gDNA extracts from sheep blood and *GAPDH* as an internal control, we found that the optimized enSfaTnpB system could detect as little as 0.5 picograms of gDNA (Supplementary Fig. S12). Finally, we used this platform to genotype 32 sheep blood samples. The results showed 100% concordance with Sanger sequencing-based genotyping results (Fig. 4K). In summary, by integrating ωRNA2.0, a split-activator strategy, and an isothermal amplification method, the enSfaTnpB system enables precise, TAM-independent discrimination of single-nucleotide variants with relatively high sensitivity and specificity, highlighting its potential for rapid, accurate SNP genotyping in clinical and agricultural settings.

### One-pot nucleic acid detection with enSfaTnpB

**Figure 5.**
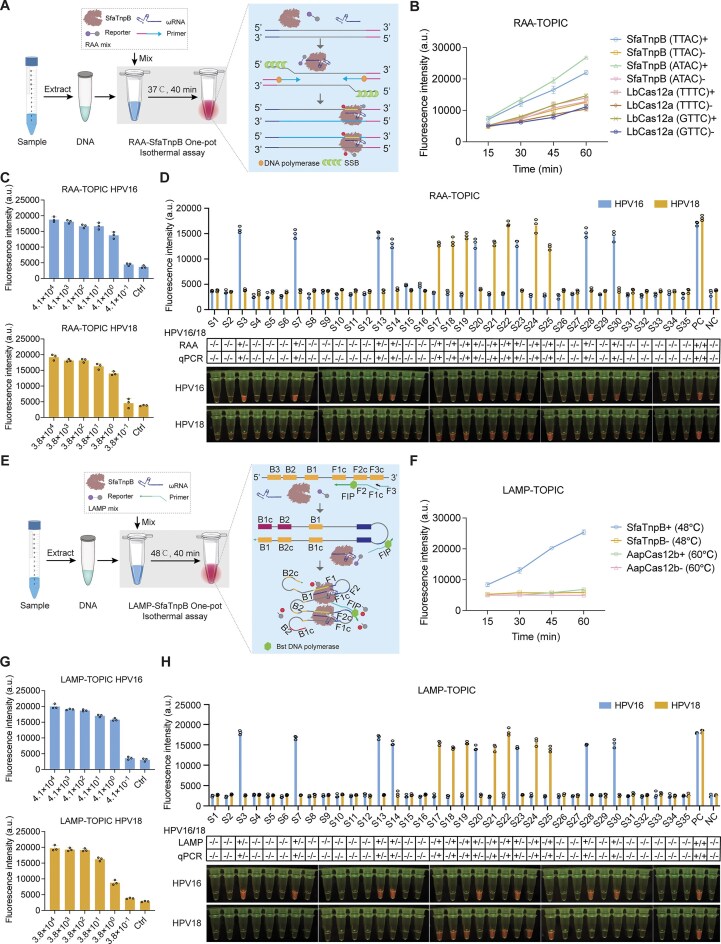
One-pot TnpB-based detection of HPV16/18. (**A**) Schematic of the workflow for RAA-TOPIC assays. (**B**) Comparison of *trans*-cleavage activity between RAA-SfaTnpB and RAA-LbCas12a in one-pot detection assays. +, presence of pMD18T-FecB plasmid; −, absence of template. (**C**) Assessment of RAA-TOPIC sensitivity using plasmids containing the *L1* gene of HPV16 or HPV18. Ctrl, no template control. (**D**) Validation of HPV16 and HPV18 detection by RAA-TOPIC in clinical samples. Reactions were incubated at 37°C for 40 min (C and D). PC, positive control containing pUC57-L1 as template; NC, no template negative control; +, positive samples; −, negative samples. (**E**) Schematic of the workflow for LAMP-TOPIC assays. (**F**) Comparison of *trans*-cleavage activity between LAMP-SfaTnpB and LAMP-AapCas12b in one-pot detection assays. +, presence of pMD18T-GAPDH plasmid; −, absence of template. (**G**) Assessment of LAMP-TOPIC sensitivity using plasmids containing the *L1* gene of HPV16 and HPV18. (**H**) Validation of HPV16 and HPV18 detection by LAMP-TOPIC in clinical samples. Reactions were incubated at 48°C for 40 min (G and H). PC, positive controls containing pUC57-L1 as a template; NC, no template negative controls; +, positive samples; −, negative samples. All data are shown as means ± s.d. (*n* = 3 technical replicates).

Leveraging isothermal amplification with the enhanced *trans*-cleavage activity of enSfaTnpB, we developed TOPIC, a one-pot nucleic acid detection platform. After initially integrating RAA to establish the RAA-TOPIC system (Fig. 5A), we validated its effectiveness using the sheep *FecB* sequence cloned into the pMD18T vector (pMD18T-FecB) as a detection target. For enSfaTnpB-based detection, ωRNAs were designed to recognize both optimal (TTAC) and suboptimal (ATAC) TAMs. Meanwhile, for comparison with LbCas12a, corresponding crRNAs targeting optimal (TTTC) and suboptimal (GTTC) PAM sequences were designed. Notably, RAA-TOPIC assays produced strong fluorescence signals with both optimal and suboptimal TAM sequences, whereas LbCas12a failed to produce significant fluorescence signals under the same conditions (Fig. 5B). These results collectively demonstrated that the TOPIC one-pot system could robustly detect target DNA.

To determine the sensitivity of the RAA-TOPIC system, we next targeted the *L1* gene of HPV16 and HPV18 using subtype-specific ωRNAs. We observed that TOPIC showed a strong signal in the presence of HPV16 (Fig. 5C, top) and HPV18 (Fig. 5C, bottom), whereas a low background signal was detected in no-template control reactions. Notably, TOPIC reached limits of detection as low as ∼4 copies/μl for both subtypes. To further evaluate its potential for clinical translation, we tested 35 samples from human HPV patients and 22 blood samples from ASFV-potentially infected swine and found complete concordance between RAA-TOPIC and qPCR results (Fig. 5D and Supplementary Figs S13 and S15). It is also worth noting that TOPIC produced sufficiently clear fluorescence signals for visualization under blue light, highlighting its suitability for field deployment in point-of-care diagnostic applications.

Given that enSfaTnpB retains high *trans*-cleavage activity at 55°C, and LAMP optimally operates between 50°C and 72°C, we further developed a LAMP-based TOPIC system (LAMP-TOPIC) (Fig. 5E). Evaluation of fluorescence and amplification performance with the above pMD18T-GAPDH target across temperatures of 50°C to 65°C in 25 μl reactions showed strong fluorescence at 50°C and 52°C. However, no ladder-like LAMP bands could be detected via gel electrophoresis. In contrast, clear LAMP bands appeared in reactions conducted at 54°C to 65°C, but no fluorescence signals were observed (Supplementary Fig. S14A and B). These results suggested that excessive amplicon accumulation at higher temperatures may suppress enSfaTnpB activity.

To investigate this temperature-related discrepancy, we conducted control fractionation experiments at 50°C using heat-inactivated LAMP amplicons as the cleavage substrate. Notably, fluorescence signals were substantially higher in reactions lacking both the LAMP primer pair and the Bst polymerase (Supplementary Fig. S14C), indicating that the presence of these components could at least partially interfere with the *trans*-cleavage activity of enSfaTnpB. To address this inhibition, we optimized the LAMP primer and Bst polymerase concentrations and ultimately found that the strongest fluorescence was obtained at combined primer volumes of 1.0–1.5 μl in a 35 μl reaction (Supplementary Fig. S14D). Further evaluation of the temperature sensitivity of the LAMP-TOPIC system using the pMD18T-GAPDH plasmid as a template across reaction temperatures of 46°C to 52°C. The results showed that 46°C and 48°C yielded the strongest fluorescence signals and the lowest detection limit (∼4 copies/μl) (Supplementary Fig. S14E). These results thus demonstrated the high sensitivity and temperature adaptability of LAMP-TOPIC assays. Additionally, for comparison, we tested AapCas12b in a one-pot LAMP system, as it reportedly performs optimally at 60°C [35]. However, AapCas12b failed to produce detectable fluorescence signals (Fig. 5F), highlighting the better compatibility of enSfaTnpB for low-temperature isothermal amplification.

Building on these findings, we further assessed the detection sensitivity of LAMP-TOPIC assays by targeting the respective *L1* genes of HPV16 and HPV18. The results demonstrated that LAMP-TOPIC could reliably detect target DNA at concentrations as low as ∼4 copies/μl (Fig. 5G), supporting its high sensitivity for low-abundance targets. To validate its sensitivity and specificity in clinical samples, we also applied LAMP-TOPIC to 35 clinical samples from HPV cases and 22 blood samples from potentially ASFV-infected swine. The results were 100% concordant with qPCR-based detection assays (Fig. 5H and Supplementary Figs S13 and S15), successfully discriminating between positive and negative samples and between HPV subtypes. These results thus confirmed the accuracy and reliability of our system with clinically relevant samples. In addition, we found that LAMP-TOPIC assays generated a bright fluorescence signal that could be readily observed with the naked eye under blue light illumination (Fig. 5H, bottom), enabling equipment-free readout and simplifying the interpretation of the results. Taken together, these results demonstrated the application of our enSfaTnpB-based one-pot nucleic acid detection platform, integrating enSfaTnpB with RAA (RAA-TOPIC) or LAMP (LAMP-TOPIC), for detecting viral DNA with high sensitivity and specificity.

### RAA-TOPIC enables high-fidelity subtype discrimination

**Figure 6.**
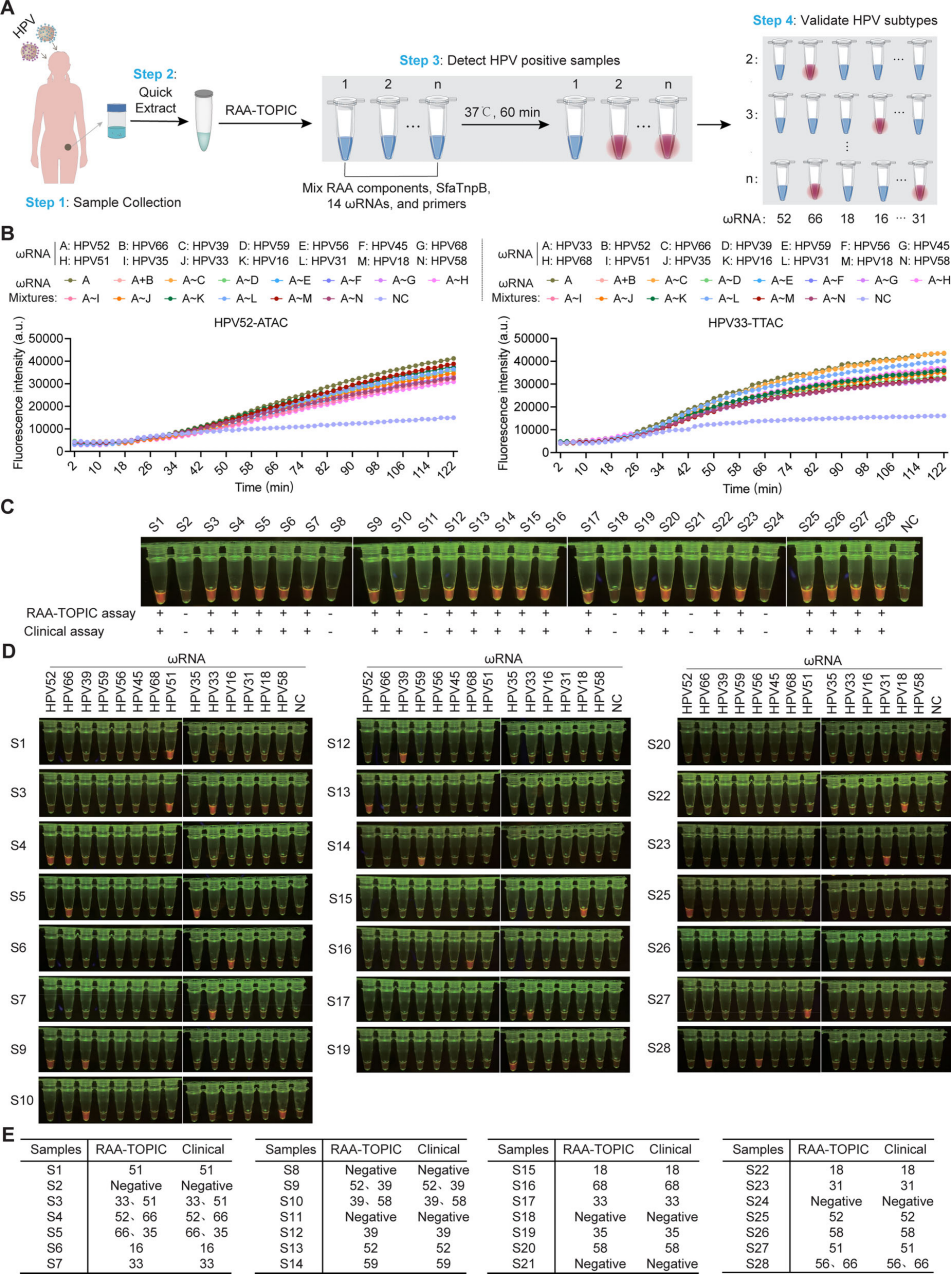
Genotyping of 14 HR-HPV subtypes using RAA-TOPIC assays. (**A**) Schematic for one-pot detection of 14 HR-HPV subtypes through RAA-TOPIC assays. (**B**) Evaluation of RAA-TOPIC tolerance for increased ωRNA diversity in a single reaction using HPV52 (TAM: 5′-ATAC) and HPV33 (TAM: 5′-TTAC) as representative targets. Data are shown as means ± s.d. (*n* = 3 technical replicates). (**C**) Validation of HR-HPV detection by RAA-TOPIC in clinical samples. +, positive samples; −, negative samples; NC, no template negative controls. (**D**) Genotyping of individual HPV-positive samples using the RAA-TOPIC assay. NC, no template negative controls. (**E**) Summary of statistical results for all tested samples.

In light of its high specificity (Fig. 2G and Supplementary Fig. S6), we further adapted the RAA-TOPIC system for one-pot subtype discrimination. For a proof-of-concept demonstration, we targeted 14 HR-HPV subtypes (52, 66, 39, 59, 56, 45, 68, 51, 35, 33, 16, 31, 18, and 58) that are strongly associated with various cancers [49]. The detection workflow comprised four steps (Fig. 6A), in which (Step 1) samples are first collected from clinical specimens, followed by (Step 2) rapid crude nucleic acid extraction. In Step 3, initial screening is performed with pooled ωRNAs in a single-tube RAA-TOPIC reaction to identify HPV-positive samples, after which (Step 4) positive samples are individually genotyped with subtype-specific ωRNAs to discriminate the precise HPV subtype. To enable subtype-specific detection, we aligned *L1* gene sequences from the 14 HPV subtypes to design subtype-specific ωRNAs (Supplementary Fig. S16). Moreover, this design strategy ensured that target sequences of each ωRNA were highly conserved among different isolates of the same subtype, thus enabling robust subtype discrimination (Supplementary Fig. S17). Specificity assays confirmed that each ωRNA showed strong specificity for its corresponding subtype, with no detectable cross-reactivity (Supplementary Fig. S18).

Given the clinical need to rapidly identify HPV-positive samples among large numbers of patients with unknown subtypes, we evaluated the system’s subtype discrimination capability by pooling all 14 subtype-specific ωRNAs into a single reaction. Fluorescence detection by plate reader showed strong, consistent signals across reactions containing 1 to 14 ωRNAs (Fig. 6B), thereby confirming that the system exhibited high tolerance to ωRNA pooling, enabling its application in subtype discrimination detection tasks. To validate this approach in a real-world context, we analyzed 28 clinical HPV samples using the RAA-TOPIC assay. To avoid primer pool complexity, we used a previously reported universal RPA primer pair containing degenerate bases that simultaneously amplify all 14 HPV subtypes [36]. Compared with current clinical detection assays, pooled RAA-TOPIC reactions accurately identified HPV-positive samples with 100% concordance (Fig. 6C). Subsequent genotyping of the HPV-positive samples with each of the 14 subtype-specific ωRNAs yielded obvious fluorescence signals under blue light (Fig. 6D) and genotyping results that were fully consistent with clinical diagnostic data (Fig. 6E). Taken together, these results establish a robust and highly specific enSfaTnpB-based platform capable of scalable, high-fidelity subtype discrimination. The TOPIC system thus represents a powerful and versatile tool well-suited for rapid genotyping and point-of-care diagnostic applications.

## Discussion

TnpB nucleases, ancestral to the Cas12 family, exhibit target-activated *trans*-cleavage activity reminiscent of Cas12a, Cas12b, and Cas12f, underscoring their potential for diagnostic detection of nucleic acids [12, 15, 50]. In this study, we identify and characterize a previously unreported TnpB ortholog, SfaTnpB, from *S. faecisuis*, which demonstrates robust *trans*-cleavage activity and relatively high single-nucleotide discrimination. Among all tested orthologs, SfaTnpB exhibited the highest *trans*-cleavage activity, comparable to that of LbCas12a and surpassing its activity at elevated temperatures, thus outperforming both reported and newly identified TnpB orthologs. We found that enSfaTnpB is compatible with isothermal amplification techniques such as RAA and LAMP and retains activity across diverse amplification buffer mixes, both of which features are essential for integration into one-pot diagnostic formats. Leveraging these properties, we developed TOPIC, a rapid and precise platform for nucleic acid detection and SNP genotyping. The RAA-TOPIC and LAMP-TOPIC platforms were further validated for clinical applications in detecting viral pathogens, ASFV, and HPV.

Because SfaTnpB showed higher *trans*-cleavage activity than other TnpB orthologs, we sought to further enhance its detection capability by modeling the SfaTnpB–ωRNA–DNA ternary complex with AlphaFold3. As the models suggested that ωRNA stem-loops might not interact with TnpB, we truncated these regions to improve RuvC domain accessibility, yielding a 105-nt ωRNA variant that markedly enhanced *trans*-cleavage activity while preserving target recognition. Further optimization of the reaction buffer and fine-tuning of the molar ratio between SfaTnpB and ωRNA yielded an enhanced system, enSfaTnpB, which provides the fundamental catalytic activity underlying TOPIC assays. Although systematic characterization of TAM preferences revealed that SfaTnpB exhibits strict dependence on a 5′-NTAC sequence, this requirement substantially limits its potential applicability for SNP genotyping. Previous work has demonstrated that splitting Cas12a crRNAs into scaffold and spacer components enables multiplexed detection of miRNAs and long RNAs by eliminating the dependence on PAM sequences [45]. Similarly, the SAHARA (Split Activator for Highly Accessible RNA Analysis) system uses split DNA and RNA fragments to trigger Cas12a activation without PAM restriction [47]. Inspired by these approaches, we tested whether SfaTnpB could tolerate analogous engineering to overcome TAM constraints. Interestingly, separating the ωRNA into distinct spacer and scaffold components completely abolished *trans*-cleavage activity, suggesting that, unlike Cas12a, TnpB requires an intact ωRNA structure for functional activation. Alternatively, a split-activator strategy preserved the robust *trans*-cleavage function of SfaTnpB while also improving its capacity to discriminate single-nucleotide mismatches and conjointly eliminating its dependence on TAM sequence. To assess its diagnostic utility, we applied the split-activator SfaTnpB system in conjunction with RAA to genotype a SNP in *FecB* associated with sheep fertility. This system achieved high fidelity in SNP discrimination, highlighting its potential for precise nucleic acid diagnostics.

At present, the incompatibility of Cas proteins with one-pot isothermal amplification, arising from the interference between *cis*-cleavage activity and amplification dynamics, has greatly limited the development and application of CRISPR-based POCT systems [16, 51]. In contrast with LbCas12a, SfaTnpB is compatible with one-pot detection assays, i.e. does not inhibit amplification by RAA or LAMP within the same reaction, thus allowing integration of either amplification strategy. This compatibility of SfaTnpB suggests a dynamic equilibrium between amplification and *trans*-cleavage. The smaller size of TnpB may reduce steric interference with DNA polymerase, although further investigation is necessary to determine the specific mechanism underlying its compatibility. Further optimization of the LAMP-SfaTnpB one-pot system resulted in greater temperature compatibility and reduced interference in the amplification process. The broad temperature tolerance of SfaTnpB also enables its integration with RAA or LAMP, which, when combined, provide rapid pathogen detection with 100% accuracy compared to current standard detection assays (e.g. qPCR). The resulting assay facilitated rapid, sensitive, and specific detection of ASFV and HPV16/18 directly from clinical samples, demonstrating its translational potential.

Although high sensitivity is a prerequisite for nucleic acid diagnostics, achieving high specificity, particularly for SNPs, typically remains challenging. CRISPR/Cas12a systems have shown limited success in this regard, in part because they cannot distinguish single-nucleotide mismatches in highly homologous regions [52, 53]. This sequence specificity is influenced by mismatch position and sequence context. Systematic profiling of SfaTnpB mismatch tolerance revealed pronounced sensitivity to mismatches at positions 5–12 proximal to the TAM, thereby enabling accurate discrimination of highly similar sequences. This feature was particularly advantageous for HPV subtyping, where extensive sequence homology in key diagnostic regions, such as the *E6, E7*, and *L1* genes, among high-risk subtypes poses a significant challenge for accurate discrimination in clinical diagnostics [54, 55]. Moreover, the limited specificity for mismatched nucleotides in Cas12a-based assays often leads to cross-reactivity and false positives [56], posing a non-trivial challenge to the accuracy of HPV subtyping in one-pot formats. In contrast, our TOPIC assay accurately subtyped 28 clinical HPV samples without cross-reactivity. We designed subtype-specific ωRNAs targeting conserved yet discriminative sequence regions associated with each HPV genotype, ensuring precise identification.

Compared to Cas12a-based systems, SfaTnpB offers distinct advantages for point-of-care diagnostics, particularly its superior single-nucleotide specificity, which enables precise discrimination of single-base mismatches and reliable detection of low-frequency variants without requiring mutation-specific crRNAs. It also exhibits robust compatibility with one-pot RAA/LAMP assays, maintaining high activity in integrated reactions where Cas12a is often inhibited, thereby facilitating true specimen-to-conclusion workflows. Additionally, the system achieves high-fidelity subtype differentiation, allowing accurate identification of closely related viral or genetic subtypes, even those differing by a single nucleotide, within complex multi-subtype samples.

Despite its promise, the enSfaTnpB-based one-pot platform has limitations. In the RAA-TOPIC, we observed a weak fluorescent background signal, likely due to nonspecific cleavage of ssDNA-FQ reporters by the RAA components. This was not observed in LAMP-TOPIC, potentially owing to differences in buffer conditions and the higher reaction temperature (48°C) of LAMP, which may suppress nonspecific cleavage. Additionally, while the split-activator strategy enabled accurate SNP genotyping, a reduction in *trans*-cleavage efficiency was noted compared to intact-target assays. Nonetheless, the SfaTnpB-based detection platform holds significant potential for optimization. Protein engineering of SfaTnpB variants and integration with advanced instrumentation could further enhance its capabilities. These improvements will expand its use in SNP genotyping, pathogen detection, and subtype differentiation. Future efforts may focus on integrating SfaTnpB into diagnostic devices for deployment in point-of-care precision medicine applications.

## Supplementary Material

gkaf1433_Supplemental_Files

## Data Availability

All additional data supporting the findings of this study are included in the Supplementary data.
