# Supplementary material for: Enhanced SfaTnpB enables single-base-specific, one-pot nucleic acid detection for high-sensitivity diagnostics
Source: Nucleic Acids Res. 2026 Jan 8;54(1):gkaf1433. doi: 10.1093/nar/gkaf1433 (PMC12781874; doi:10.1093/nar/gkaf1433)
Supplement: gkaf1433_Supplemental_Files [file gkaf1433_supplemental_files.zip › Supplementary Figures.pdf]

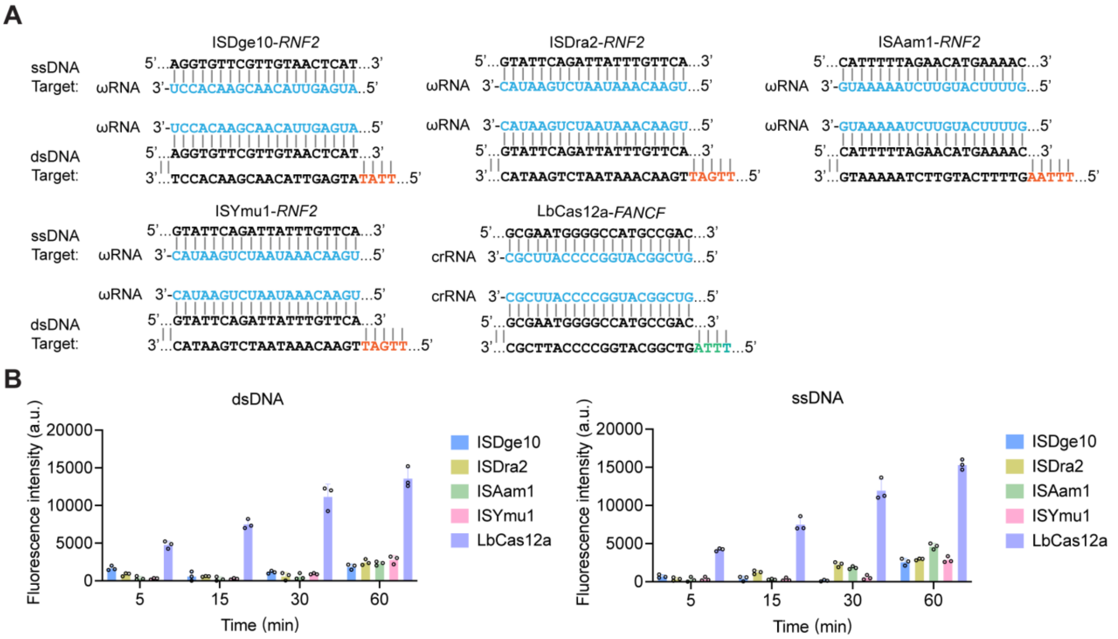

Figure S1. Evaluation of *trans*-cleavage activity of TnpB orthologs and LbCas12a. (A) Sequences of target ssDNAs and dsDNAs with their corresponding guide RNAs. Guide RNA sequences are indicated in blue; TAM sequences for TnpB orthologs are shown in orange; PAM sequence for LbCas12a is displayed in green. (B) Evaluation of *trans*-cleavage activity of ISDra2, ISDge10, ISAam1, and ISYmu1 TnpBs, as well as LbCas12a, using an ssDNA-FQ reporter assay. All data are shown as means ± s.d. (n = 3 technical replicates).

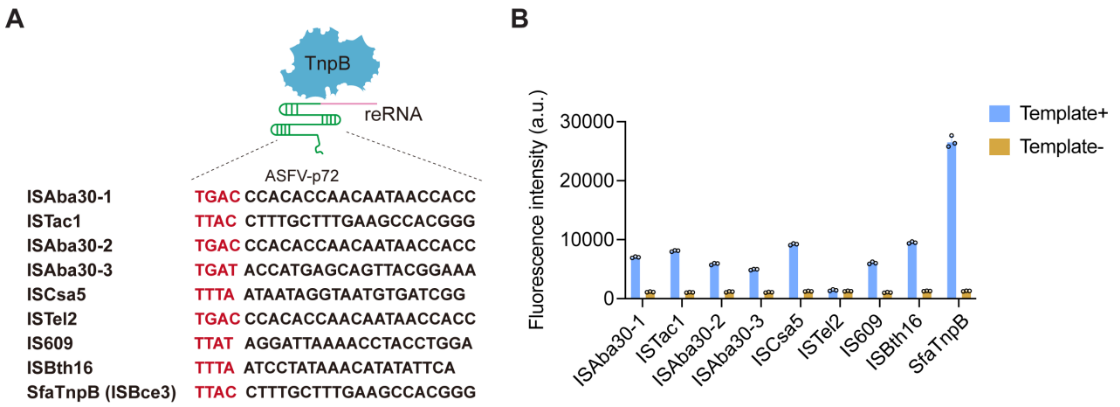

Figure S2. Evaluation of *trans*-cleavage activity for nine newly identified TnpB orthologs. (A) Schematic of the nine newly identified TnpB orthologs. Predicted TAM sequences are highlighted in red; ωRNA guide sequences are shown in black. (B) Assessment of *trans*-cleavage activity of each TnpB ortholog using dsDNA activators. All data are shown as means ± s.d. (*n* = 3 technical replicates).

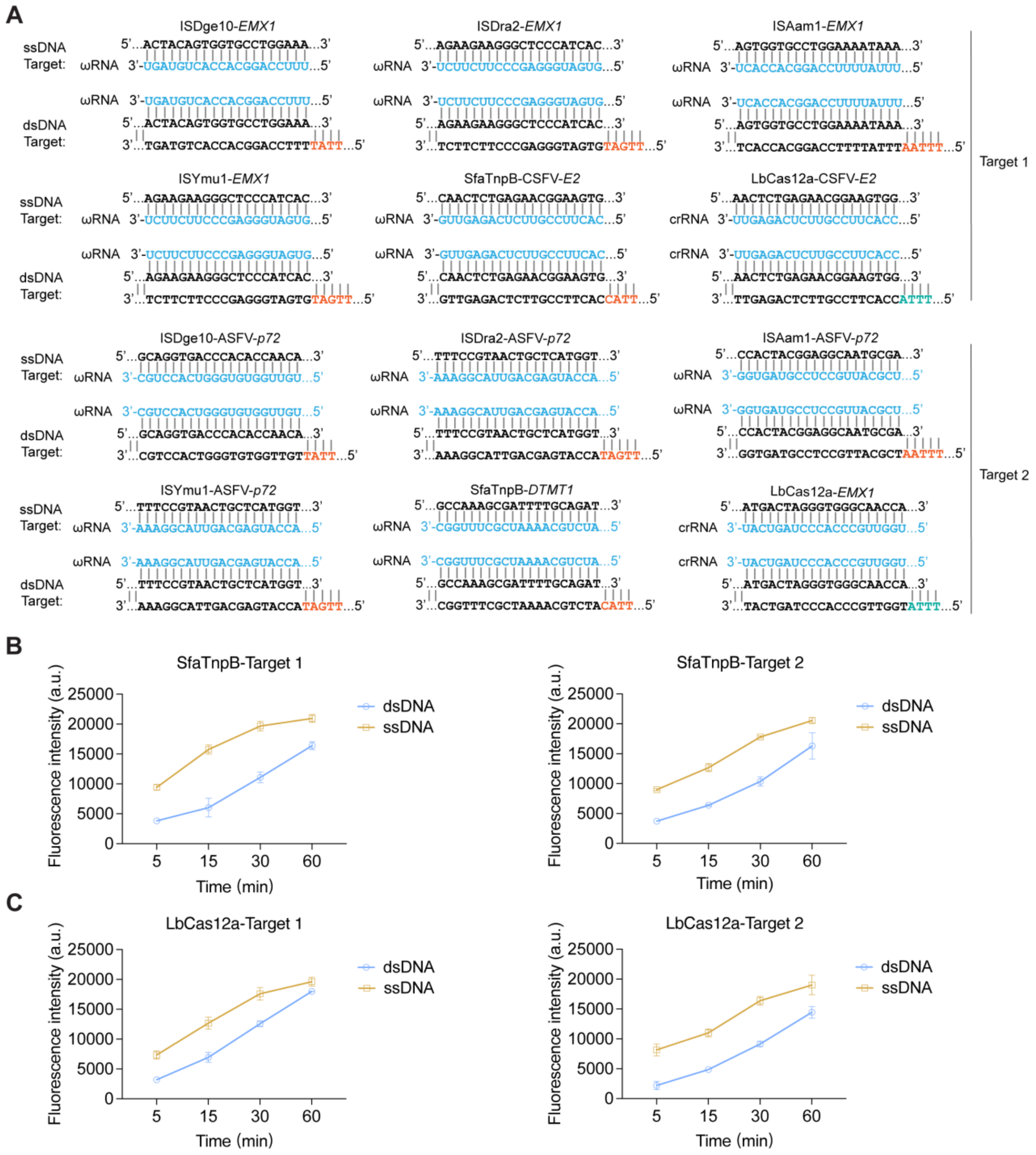

Figure S3. Evaluation of *trans*-cleavage activity for SfaTnpB, other TnpB orthologs, and LbCas12a. (A) Sequences of target ssDNAs and dsDNAs with their corresponding guide RNAs. Guide RNA sequences are indicated in blue; TAM sequences for TnpB orthologs are shown in orange; PAM sequence for LbCas12a is displayed in green. (B) Comparison of SfaTnpB *trans*-cleavage activity between ssDNA and dsDNA targets. (C) Comparison of LbCas12a *trans*-cleavage activity between ssDNA and dsDNA targets. All data are shown as means ± s.d. (n = 3 technical replicates).

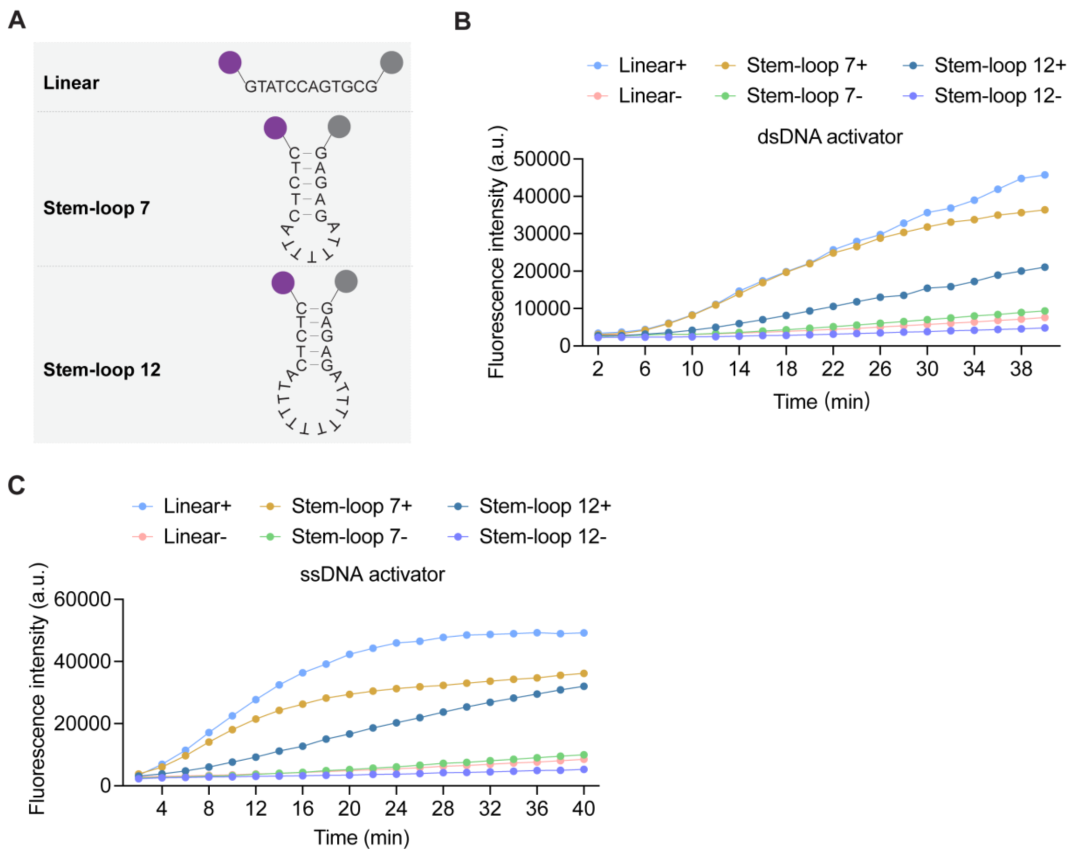

Figure S4. Evaluation of SfaTnpB *trans*-cleavage activity on ssDNA-FQ reporters containing stem-loop structures. (A) Schematic of ssDNA reporters with stem-loop structures. (B and C) Assessment of SfaTnpB *trans*-cleavage activity on stem-loop-containing reporters with dsDNA (B) and ssDNA (C) activators. All data are shown as means ± s.d. (n = 3 technical replicates).

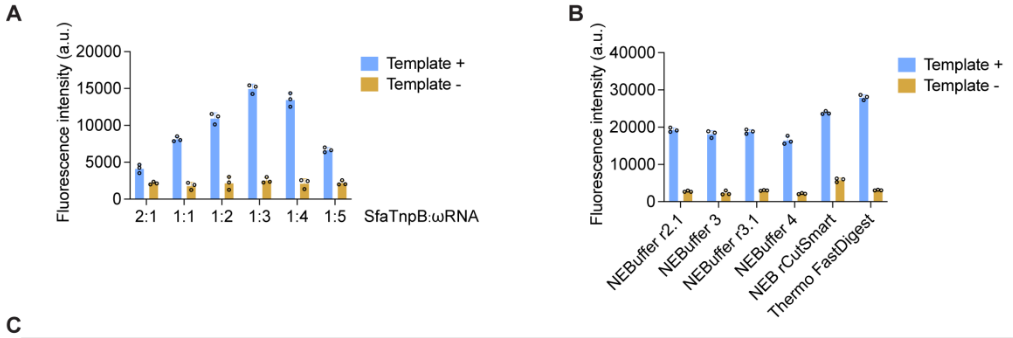

C

Reaction Buffer	NEBuffer™ r2.1	NEBuffer™ 3	NEBuffer™ r3.1	NEBuffer™ 4	rCutSmart™ Buffer	Thermo FastDigest
Component	50 mM NaCl<br>10 mM Tris-HCl<br>10 mM MgCl2<br>100 µg/ml Recombinant Albumin<br>pH 7.9@25°C	100 mM NaCl<br>50 mM Tris-HCl<br>10 mM MgCl2<br>1 mM DTT<br>pH 7.9@25°C	100 mM NaCl<br>50 mM Tris-HCl<br>10 mM MgCl2<br>100 µg/ml Recombinant Albumin<br>pH 7.9@25°C	50 mM Potassium Acetate<br>20 mM Tris-acetate<br>10 mM Magnesium Acetate<br>1 mM DTT<br>pH 7.9@25°C	50 mM Potassium Acetate<br>20 mM Tris-acetate<br>10 mM Magnesium Acetate<br>100 µg/ml Recombinant Albumin<br>pH 7.9@25°C	Unknown

Figure S5. Optimization of SfaTnpB *trans*-cleavage reaction components. (A) Optimization of the SfaTnpB to ωRNA molar ratio. (B) Screen of commercially available reaction buffers for *trans*-cleavage activity. (C) Detailed composition of all tested buffer systems. All data are shown as means ± s.d. (*n* = 3 technical replicates).

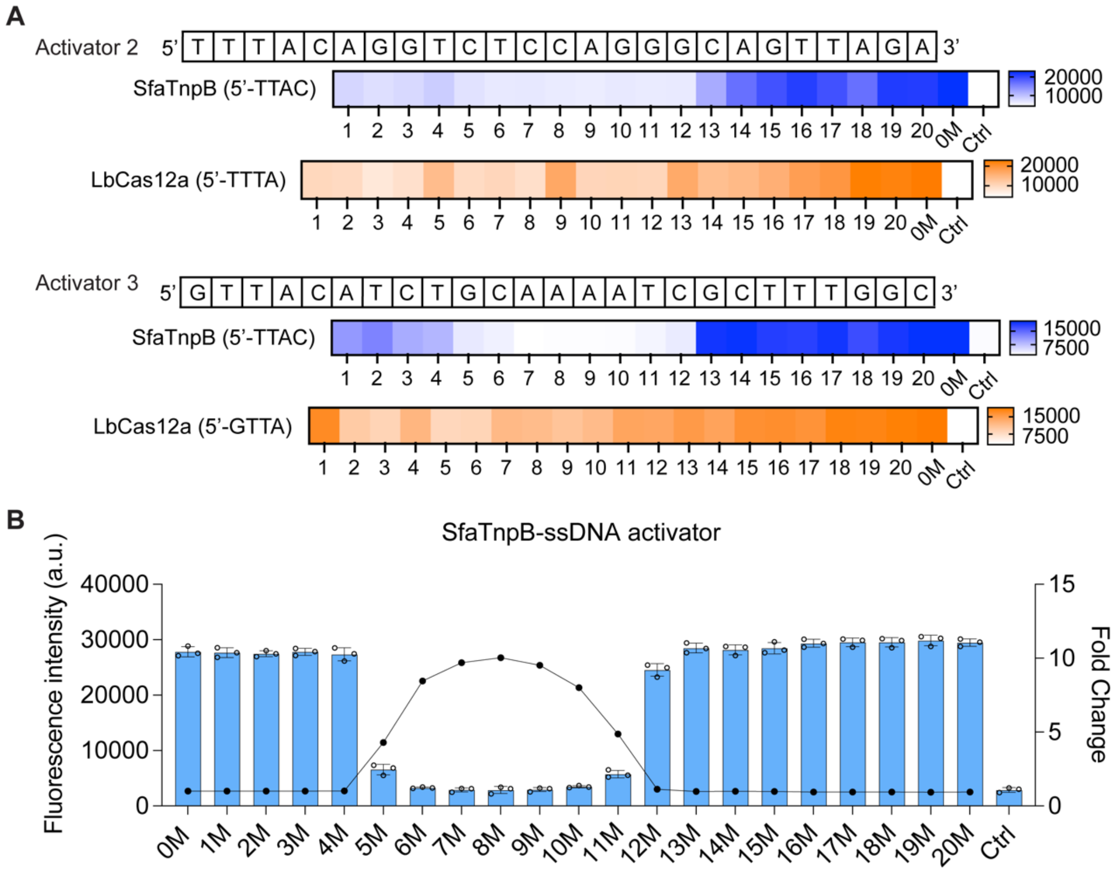

Figure S6. Assessment of single-base discrimination specificity of SfaTnpB and LbCas12a. (A) Heat maps showing single-nucleotide discrimination by SfaTnpB and LbCas12a with two different dsDNA activators. (B) Assessment of single-nucleotide discrimination by SfaTnpB and LbCas12a using ssDNA activators. 0M, fully matched target; Ctrl, no template control. All data are shown as means ± s.d. (*n* = 3 technical replicates).

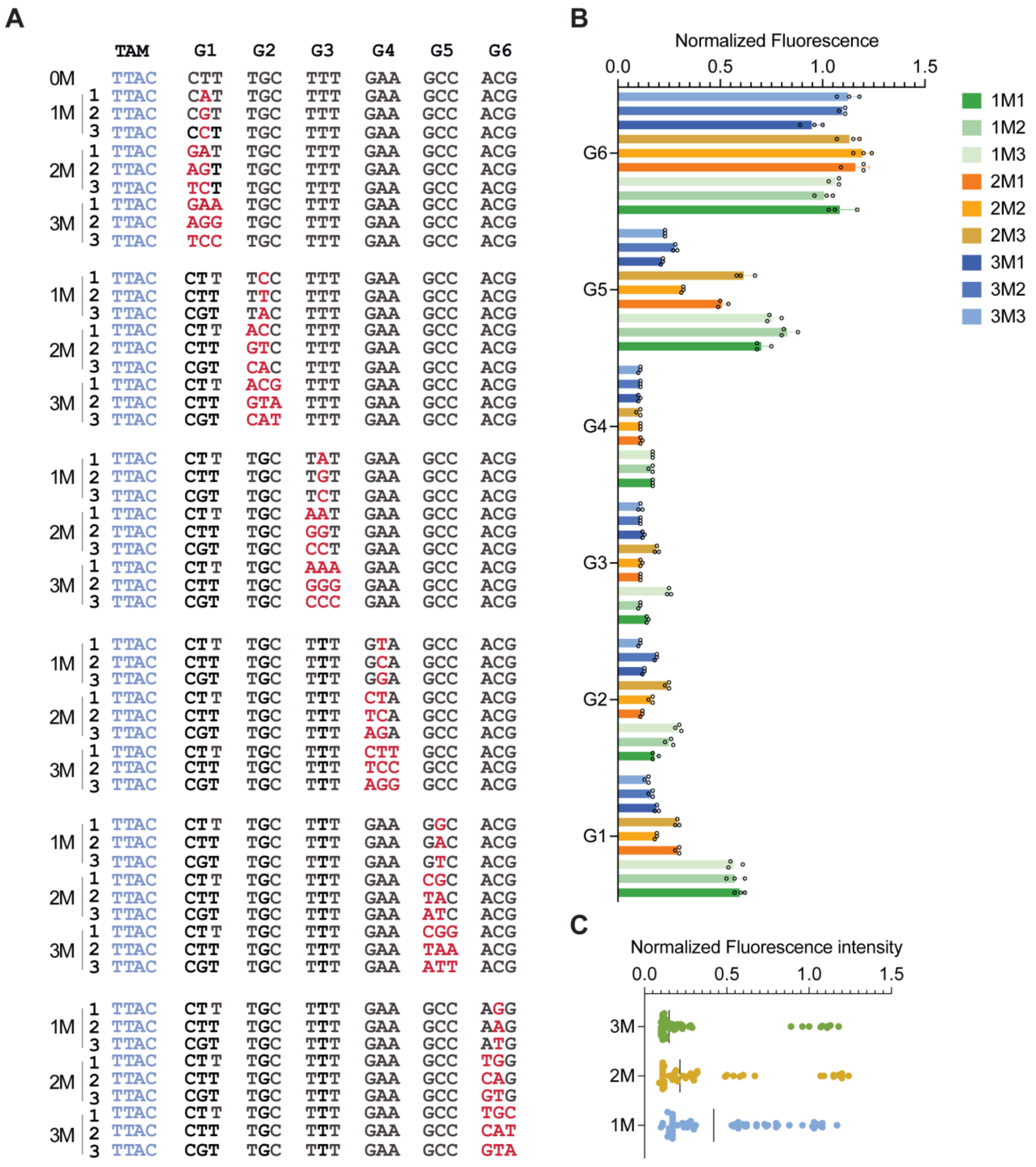

Figure S7. Evaluation of the effects of mismatch position and type on SfaTnpB specificity. (A) Schematic representation of the mismatch profiles. 0M indicates a fully matched target; 1M, 2M, and 3M represent targets containing 1, 2, or 3 nucleotide mismatches, respectively. (B) Normalized fluorescence signals for individual mismatch profiles. (C) Average normalized fluorescence signals across mismatch categories. All data are shown as means ± s.d. (n = 3 technical replicates).

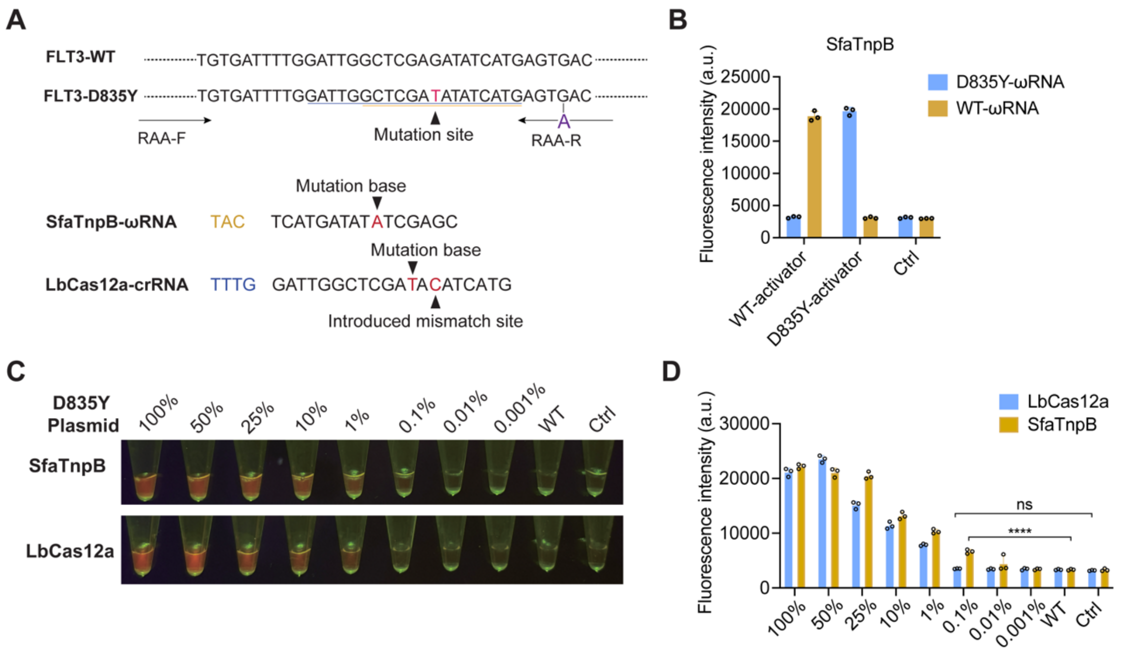

Figure S8. Comparison of detection limits for variant allele frequencies (VAFs) between RAA-SfaTnpB and RAA-Cas12a. (A) Sequences of the D835Y-targeting crRNA for Cas12a, the corresponding ωRNA for SfaTnpB, and the *FLT3* wild-type (WT) and D835Y mutant regions. The D835Y mutation involves a G-to-T substitution. (B) Specificity analysis of the D835Y-targeting ωRNA. (C and D) Sensitivity comparison of RAA-SfaTnpB and RAA-Cas12a using serial gradients of D835Y variant allele frequencies, assessed by visual detection under blue light (C) and by measuring fluorescence intensity (D). Ctrl, no template control. All data are shown as means ± s.d. (n = 3 technical replicates).

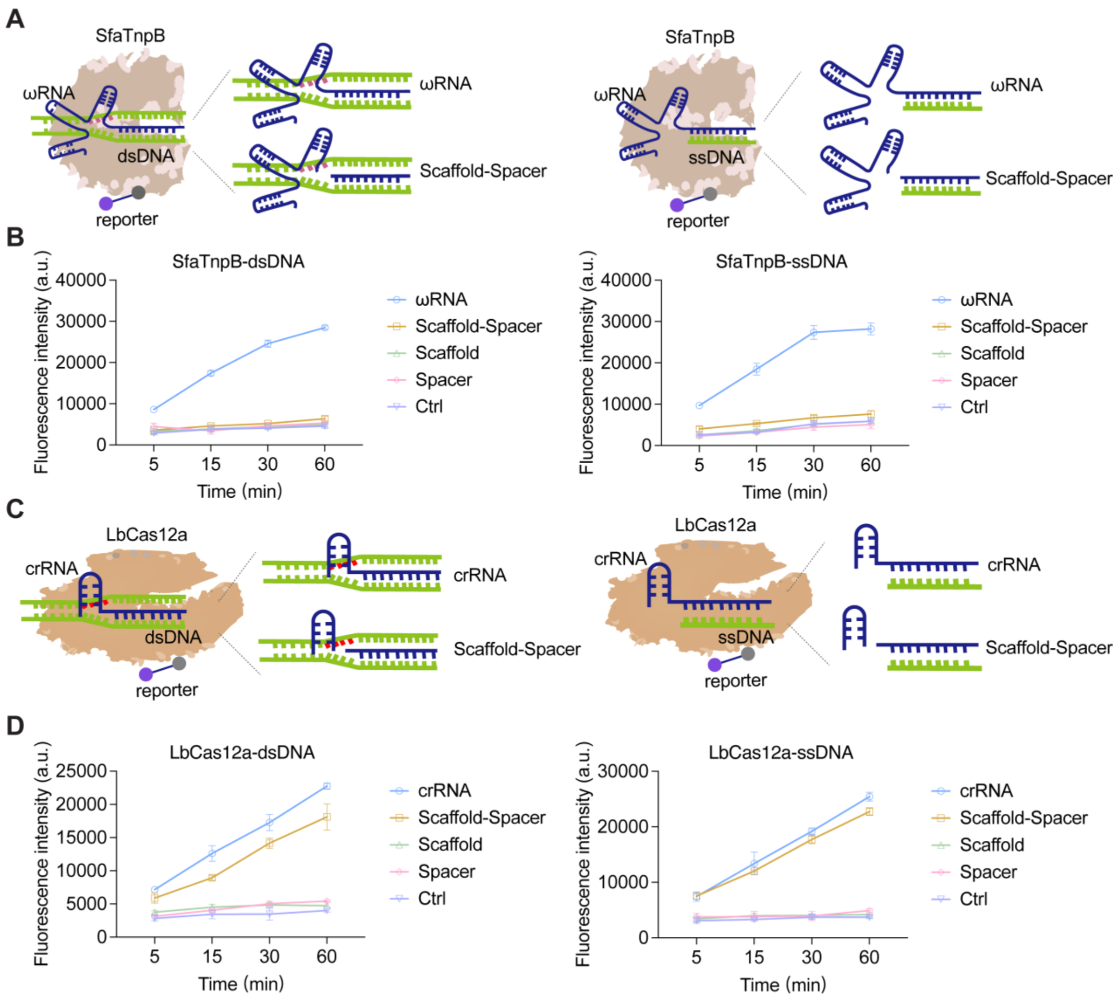

Figure S9. Split and full-length ωRNA (crRNA)-directed *trans*-cleavage activity of SfaTnpB and LbCas12a. (A) Schematic of split and full-length ωRNA-directed *trans*-cleavage activity of SfaTnpB. (B) Assessment of split and full-length ωRNA-directed *trans*-cleavage activity of SfaTnpB with ssDNA or dsDNA targets. Ctrl, no template control. Data are shown as means ± s.d. (n = 3 technical replicates). (C) Schematic of split crRNA and full-length crRNA-directed *trans*-cleavage activity of LbCas12a. (D) Assessment of split and full-length crRNA-directed *trans*-cleavage activity of LbCas12a with ssDNA or dsDNA targets. Ctrl, no template control. All data are shown as means ± s.d. (n = 3 technical replicates).

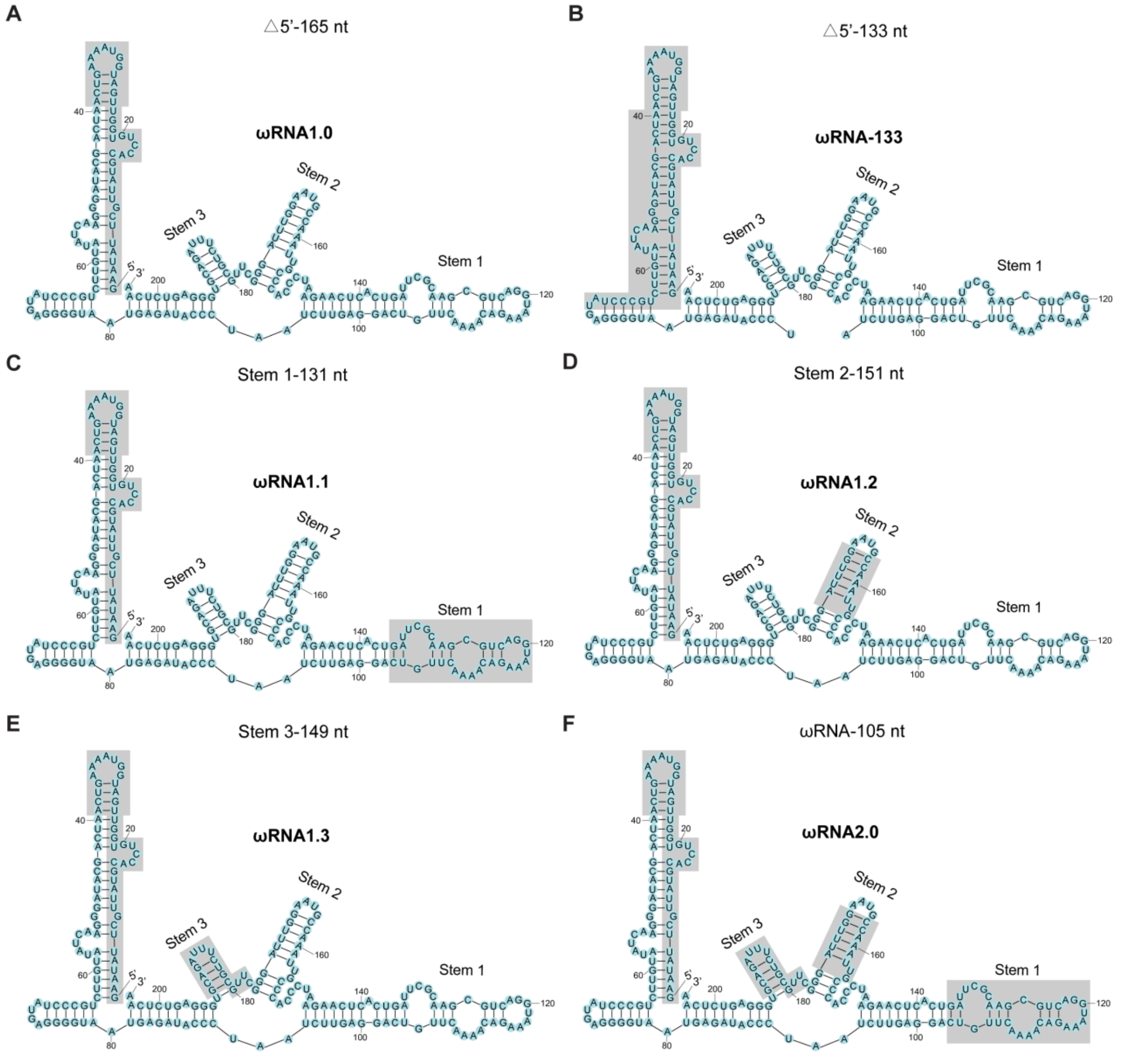

Figure S10. Engineering of SfaTnpB ωRNA. (A) Predicted secondary structure of ωRNA1.0 with 5' region truncation. (B) Predicted secondary structure of ωRNA-133 with 5' region truncation. (C). Predicted secondary structure of ωRNA1.1 with truncation of the 5' region and Stem 1. (D) Predicted secondary structure of ωRNA1.2 with truncation of the 5' region and Stem 2. (E) Predicted secondary structure of ωRNA1.3 with truncation of the 5' region Stem 3. (F) Predicted secondary structure of ωRNA2.0 with combined truncations of the 5' region, Stem 1, Stem 2, and Stem 3. Truncated areas are highlighted in grey boxes.

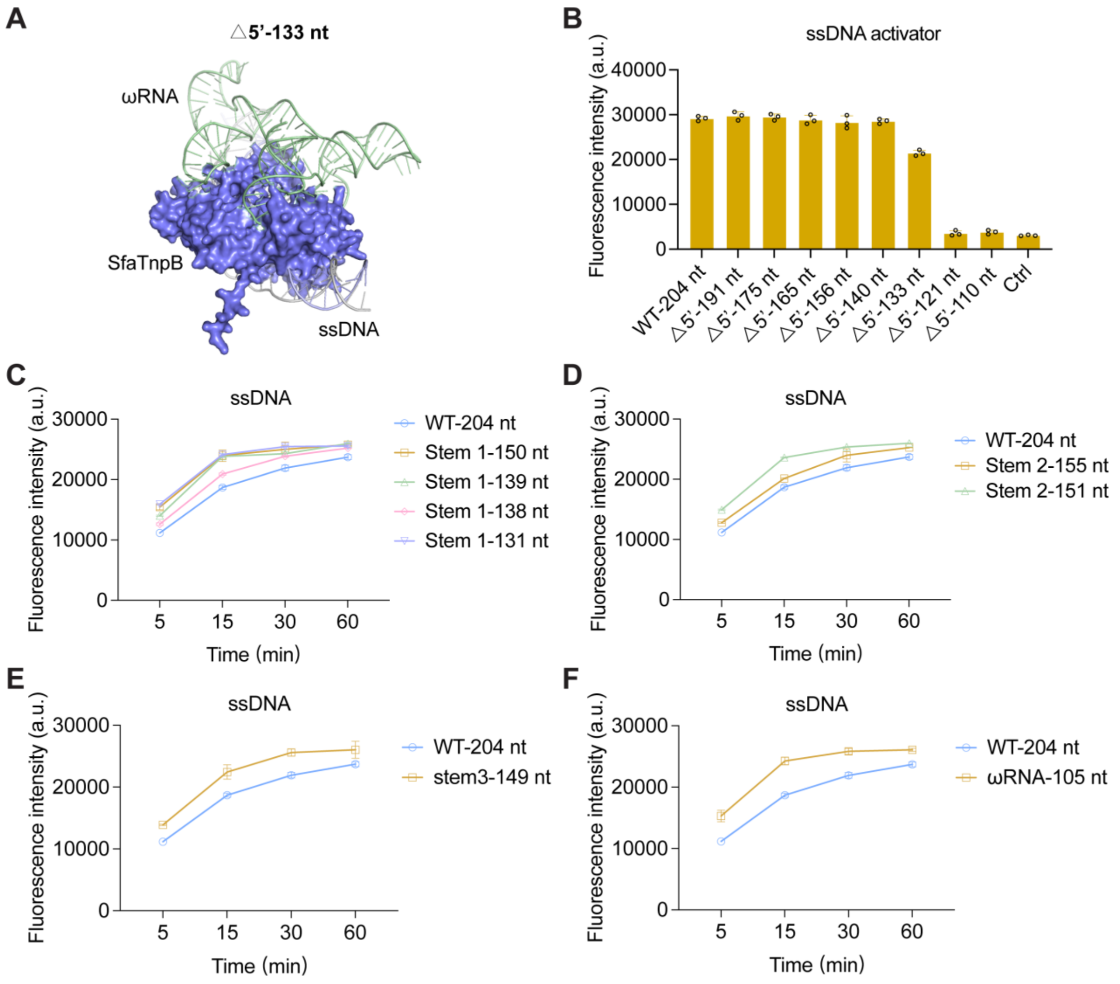

Figure S11. Engineering of SfaTnpB ωRNA improved *trans*-cleavage activity using ssDNA activator. (A) Predicted structures of the SfaTnpB-ssDNA-ωRNA complexes, generated by AlphaFold3. (B) Assessment of *trans*-cleavage activity using ωRNAs with progressive 5′ truncation and ssDNA activators. Ctrl, no template control. (C to E). Evaluation of the *trans*-cleavage activity by ωRNAs with truncated Stem1 (C), Stem2 (D), or Stem3 (E) regions using a ssDNA activator. (F) Comparison of SfaTnpB *trans*-cleavage activity using native ωRNA (WT-ωRNA) versus ωRNA2.0 with a ssDNA activator. All data are shown as means ± s.d. (n = 3 technical replicates).

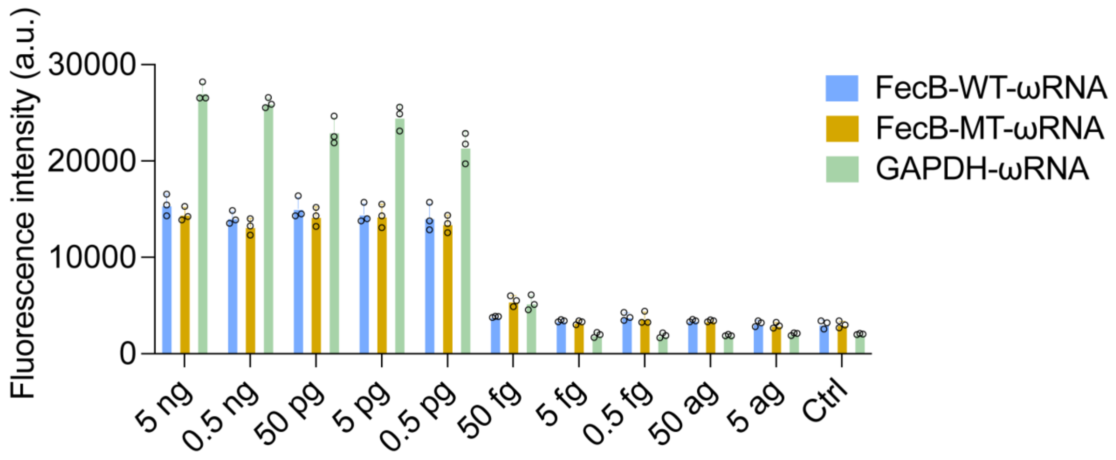

Figure S12. Evaluation of the sensitivity of the enSfaTnpB-based SNP detection system. Ctrl, no template control. Data are shown as means ± s.d. (*n* = 3 technical replicates).

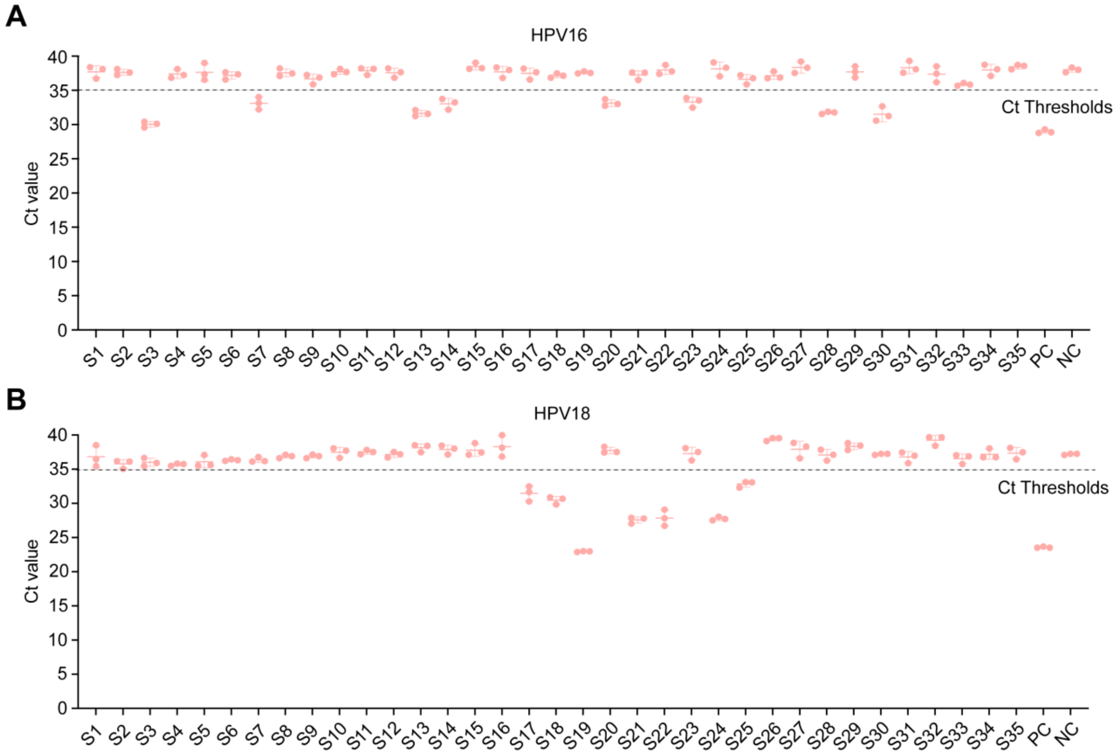

Figure S13. Detection of 35 HPV clinical samples using qPCR. (A) HPV16 clinical samples are validated by qPCR. (B) HPV18 clinical samples are validated by qPCR. PC, positive control containing pUC57-L1 as template; NC, no template negative control.

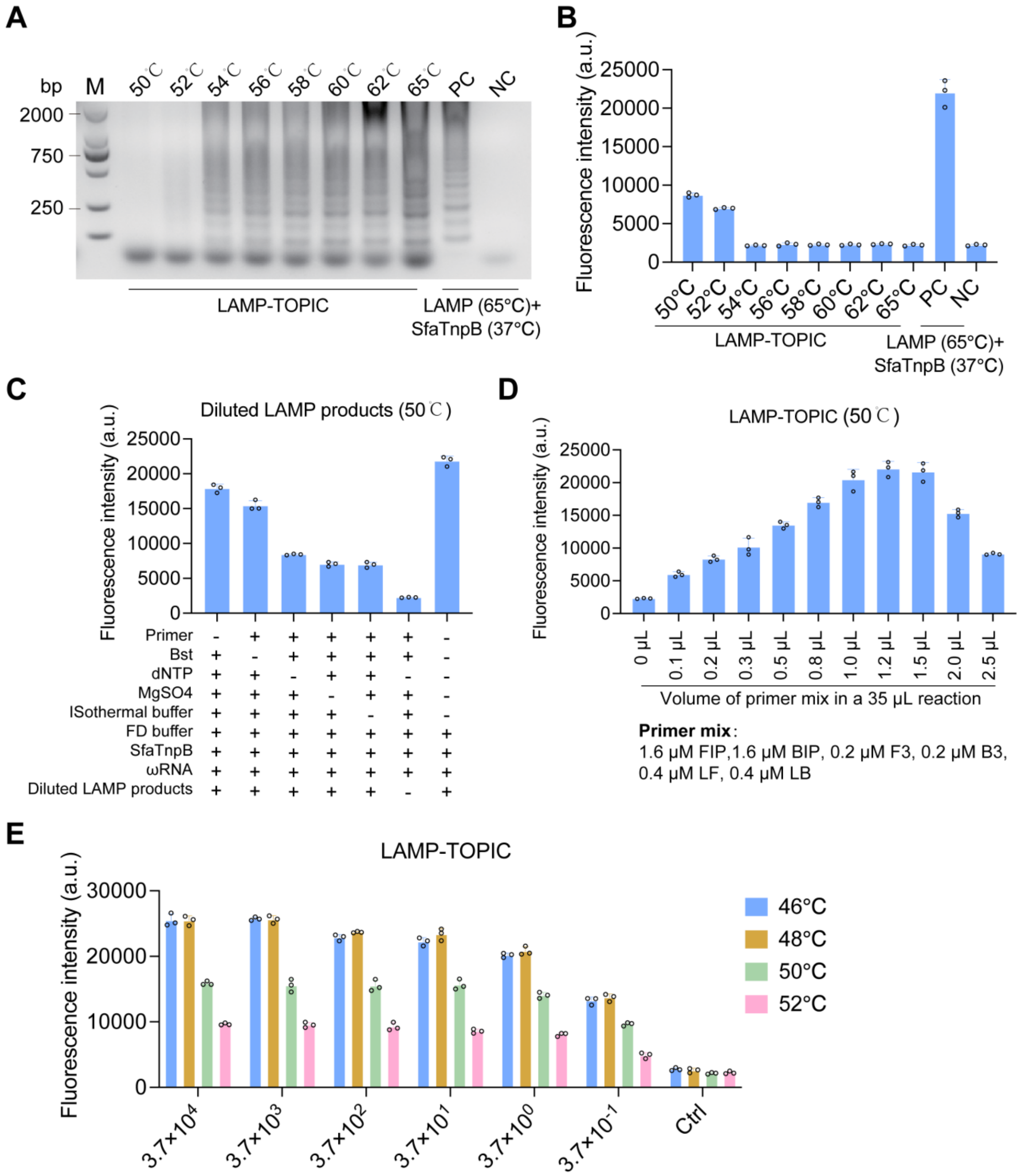

Figure S14. Optimization of the LAMP-TOPIC system. (A) Agarose gel electrophoresis showing LAMP amplification products at the indicated temperature. A two-step protocol was used as reference, consisting of LAMP amplification followed by the addition of SfaTnpB and ωRNA. PC, positive control containing pMD18T-GAPDH as template; NC, no template negative control. (B) Assessment of LAMP-TOPIC *trans*-cleavage activity at the indicated temperatures. (C) Evaluation of component requirements for LAMP-TOPIC using control fractionation experiments. (D) Optimization of LAMP primers and Bst polymerase concentrations.

(E) Evaluation of the sensitivity of the LAMP-TOPIC assay at different temperatures. Ctrl, no template control. All data are shown as means ± s.d. (n = 3 technical replicates).

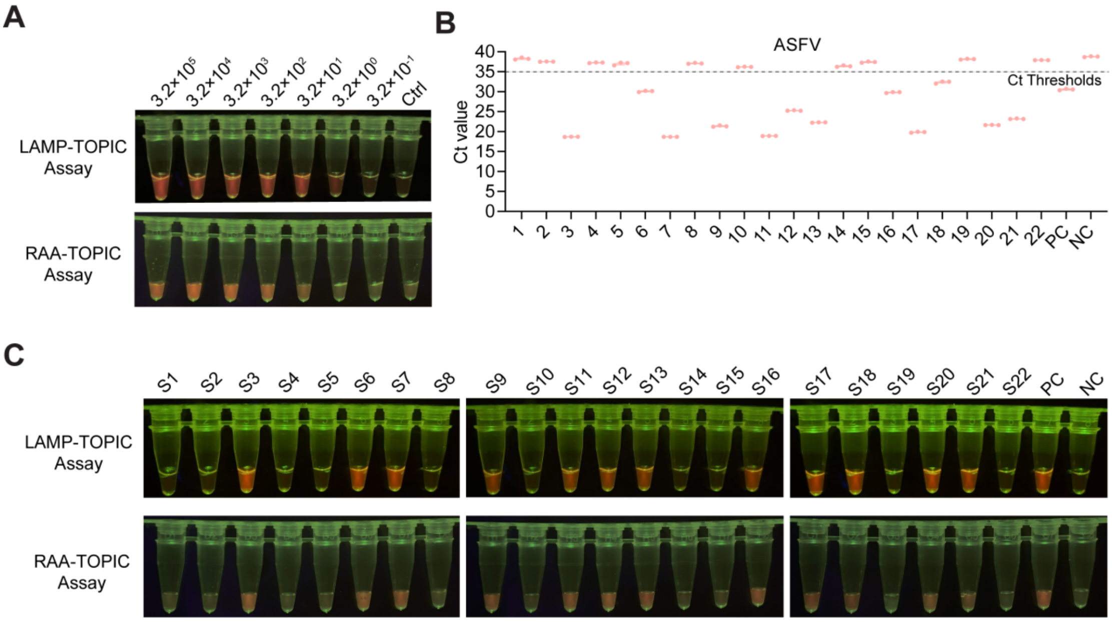

Figure S15. Validation of ASFV clinical samples using the LAMP-TOPIC and RAA-TOPIC assay. (A) Evaluation of the sensitivity of LAMP-TOPIC and RAA-TOPIC for the detection of the ASFV *p72* gene by using the pUC57-p72 plasmid. (B) Detection of 22 ASFV clinical samples using qPCR. (C) Detection of 22 ASFV clinical samples using the LAMP-TOPIC and RAA-TOPIC assay. Fluorescence signals were clearly visible under blue light. PC, positive control containing pUC57-p72 as template; NC, no template negative control.

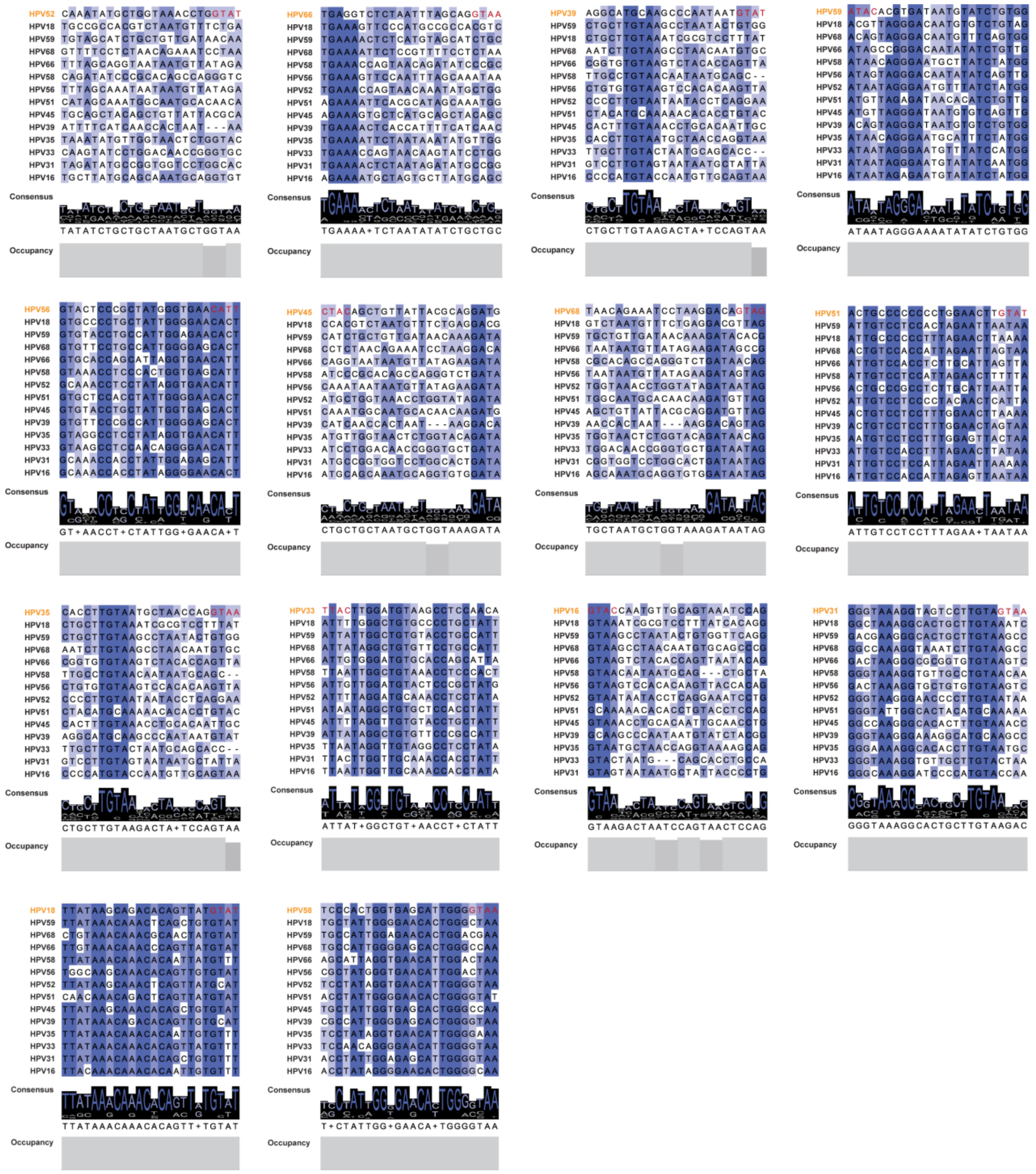

Figure S16. The specificity of 14 HR-HPV subtype-specific ωRNAs was validated by sequence alignment with Jalview software. HPV subtypes are shown in yellow. TAM sequences are highlighted in red.

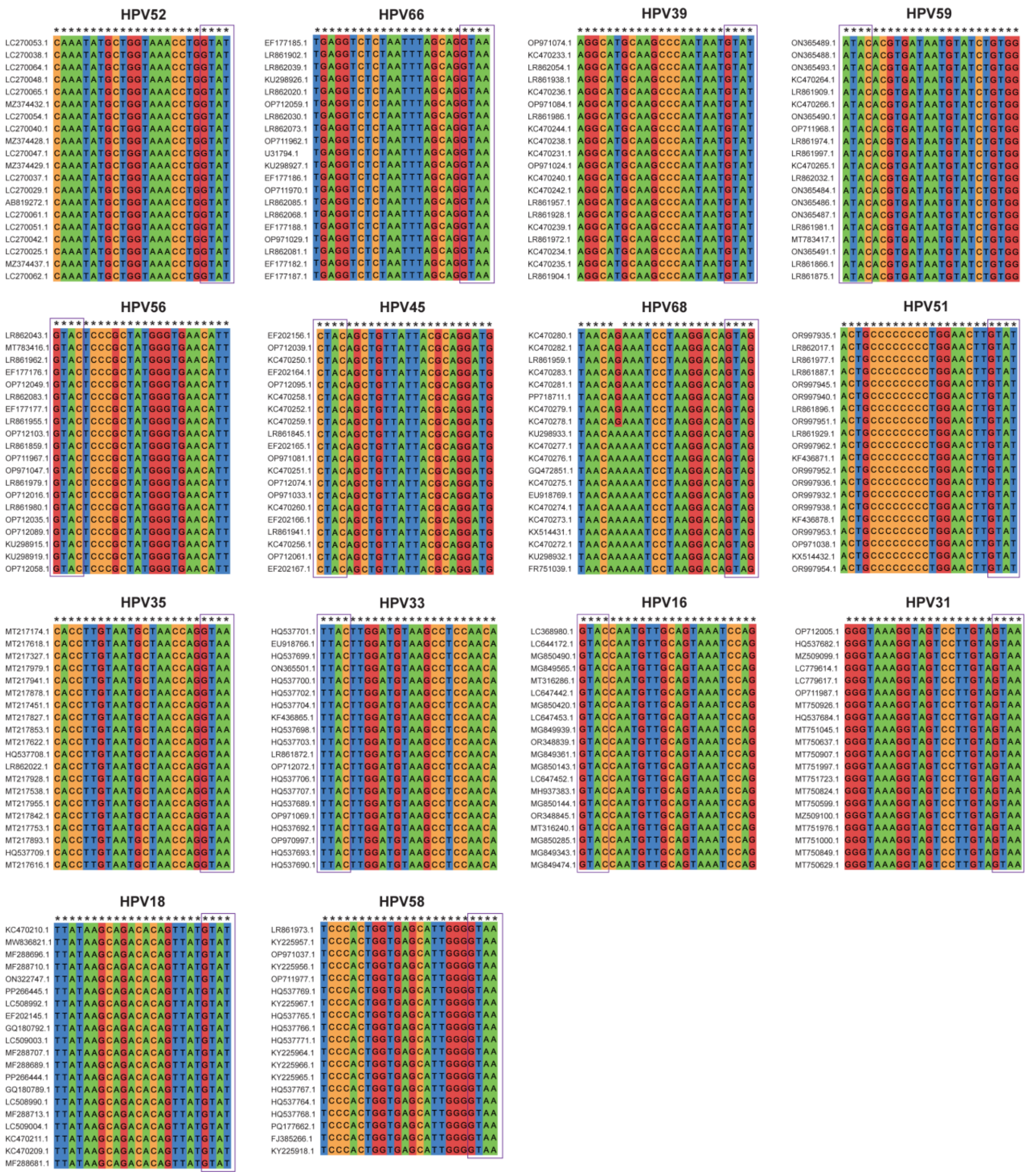

Figure S17. Conservation analysis of 14 HR-HPV subtype-specific ωRNAs among different isolates of the same subtype. Alignments were performed with ClustalX2 software. Perfectly matched nucleotides are indicated with asterisks, and TAM sequences are highlighted with boxes.

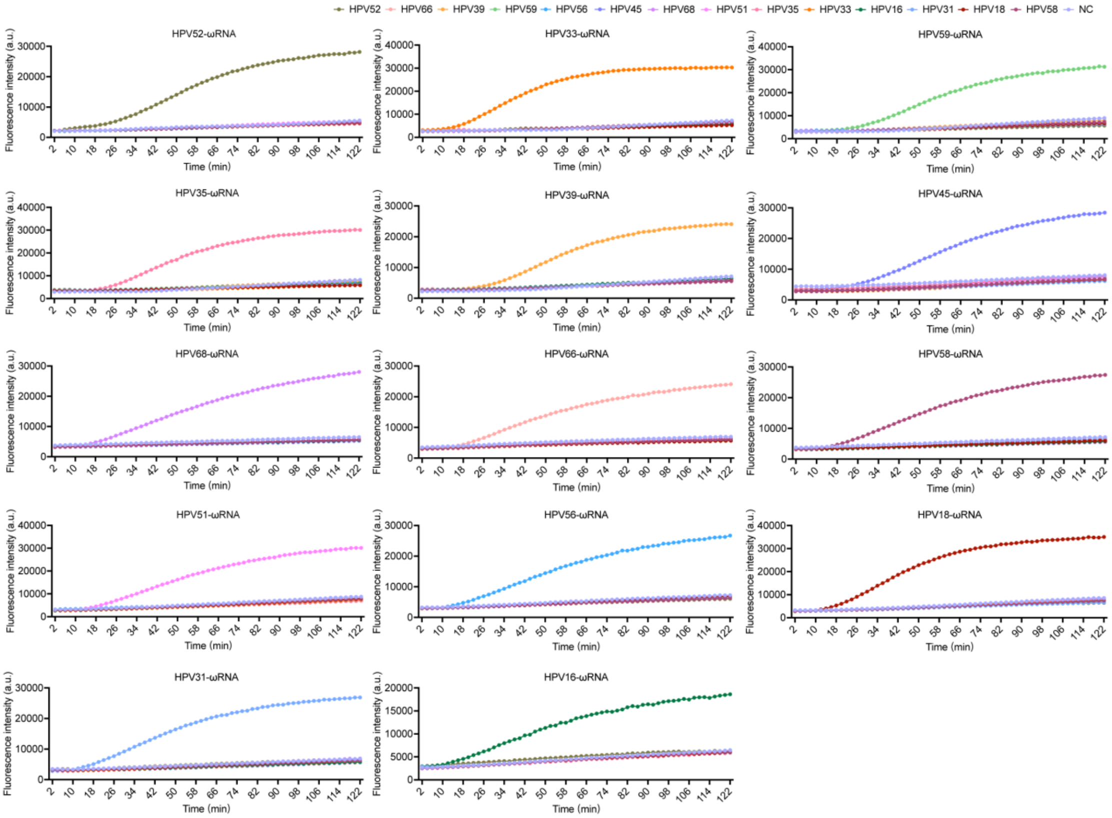

Figure S18. Specificity assessment of 14 HR-HPV subtype-specific ωRNAs using ssDNA-FQ reporter assay.
